# Offshore marine actinopterygian assemblages from the Maastrichtian–Paleogene of the Pindos Unit in Eurytania, Greece

**DOI:** 10.7717/peerj.10676

**Published:** 2021-01-20

**Authors:** Thodoris Argyriou, Donald Davesne

**Affiliations:** 1 UMR 7207 (MNHN—Sorbonne Université—CNRS) Centre de Recherche en Paléontologie, Museum National d’Histoire naturelle, Paris, France; 2 Department of Earth Sciences, University of Oxford, Oxford, UK; 3 UMR 7205 (MNHN—Sorbonne Université—CNRS—EPHE), Institut de Systématique, Évolution, Biodiversité, Museum National d’Histoire naturelle, Paris, France

**Keywords:** Actinopterygii, Fossil fishes, Maastrichtian, Paleocene, Greece, Tethys, K–Pg Extinction, Pindos Unit

## Abstract

The fossil record of marine ray-finned fishes (Actinopterygii) from the time interval surrounding the Cretaceous–Paleogene (K–Pg) extinction is scarce at a global scale, hampering our understanding of the impact, patterns and processes of extinction and recovery in the marine realm, and its role in the evolution of modern marine ichthyofaunas. Recent fieldwork in the K–Pg interval of the Pindos Unit in Eurytania, continental Greece, shed new light on forgotten fossil assemblages and allowed for the collection of a diverse, but fragmentary sample of actinopterygians from both late Maastrichtian and Paleocene rocks. Late Maastrichtian assemblages are dominated by Aulopiformes (†Ichthyotringidae, †Enchodontidae), while †Dercetidae (also Aulopiformes), elopomorphs and additional, unidentified teleosts form minor components. Paleocene fossils include a clupeid, a stomiiform and some unidentified teleost remains. This study expands the poor record of body fossils from this critical time interval, especially for smaller sized taxa, while providing a rare, paleogeographically constrained, qualitative glimpse of open-water Tethyan ecosystems from both before and after the extinction event. Faunal similarities between the Maastrichtian of Eurytania and older Late Cretaceous faunas reveal a higher taxonomic continuum in offshore actinopterygian faunas and ecosystems spanning the entire Late Cretaceous of the Tethys. At the same time, the scarcity of Paleocene findings offers tentative clues for a depauperate state of Tethyan ichthyofaunas in the aftermath of the K–Pg Extinction.

## Introduction

The mass extinction event at the K–Pg boundary had a devastating impact on global marine ecosystems, causing the complete extinction and replacement of many staple macrofaunal components of the Mesozoic. Marine ray-finned fish (Actinopterygii) communities—especially pelagic ones—were heavily restructured by this extinction event, with many long-lasting lineages (e.g., †pachycormids, †ichthyodectiforms, epipelagic aulopiforms) selectively meeting their end, or becoming decimated, at, or near, the end of the Cretaceous (Cavin, 2002; Friedman, 2009; Friedman et al., 2010; Sibert & Norris, 2015; Guinot & Cavin, 2016). At the same time, the ecological space freed by the K–Pg Extinction is thought to have enabled the staged ecological diversification of phylogenetically disparate lineages during the Paleogene, and the subsequent dominance of the hyper-diverse acanthomorph teleosts in pelagic marine habitats (Friedman, 2010; Near et al., 2012; Miya et al., 2013; Near et al., 2013; Betancur-R, Ortí & Pyron, 2015; Guinot & Cavin, 2016; Alfaro et al., 2018; Capobianco, Foreman & Friedman, 2019; Friedman et al., 2019; Capobianco et al., 2020). Such trends become apparent in broad overviews of the Cretaceous–Paleogene fossil record of actinopterygians (Cavin, 2002; Friedman, 2009; Guinot & Cavin, 2016), and receive further support from implications of molecular time scales (Near et al., 2012; Near et al., 2013; Betancur-R, Ortí & Pyron, 2015; Alfaro et al., 2018; Friedman et al., 2019), or ichthyolith (Arambourg, 1952; Becker, Mallery & Chamberlain, 2010; Sibert, Hull & Norris, 2014; Sibert & Norris, 2015) and otolith data (Schwarzhans, 1996, 2004; Schwarzhans & Milàn, 2017). However, assemblages containing taxonomically and phylogenetically informative marine fossils in the form of articulated or semi-articulated skeletons from the ~16 Myr interval (Maastrichtian–Paleocene) surrounding the actual extinction boundary are rare. This virtual gap of the actinopterygian body fossil record poses obstacles for addressing key biodiversity issues, such as the pace of extinction and recovery, the status of biodiversity and ecosystems prior to, and immediately after the extinction, as well as the pattern and timing of major actinopterygian radiations.

The recent discovery of Maastrichtian fish-bearing exposures of the Pindos Unit in Gavdos Island (Cavin, Alexopoulos & Piuz, 2012) highlighted the paleontological potential of alpine sedimentary successions exposed over 100 km^2^ in Greece, and rekindled scientific interest in the study of penecontemporaneous horizons exposed in continental Greece. Maastrichtian–Paleogene (largely Paleocene) articulated and semi-articulated fish-bearing horizons, contained within the nappes of the largely pelagic Pindos Unit in Eurytania, Aetolia (both in continental Greece) and the Central Peloponnese, have been known to geologists and private collectors since at least the 1950s and 1960s (Renz, 1955; Koch & Nicolaus, 1969; Kallergis, Koch & Nicolaus, 1970), but have never been subjected to systematic collecting or study to date, and fell into obscurity soon after their discovery. Previous information on the Maastrichtian–Paleocene fish assemblages of continental Greece comes from a poorly circulated work on the geology of the region of Eurytania, in which fossil fishes are only a minor component, and are presented in the form of preliminary faunal lists—containing several taxonomical errors and redundancies—and a handful of figures (Koch & Nicolaus, 1969). The authors reported on several fossiliferous localities in Eurytania, which span the K–Pg Boundary. The previously published faunal lists (unedited) include five chondrichthyan genera (*Hexanchus*; *Isurus*; †*Corax*; *Odontaspis*; *Squalus*) and seven or eight actinopterygian genera (†*Holcolepis*; †*Osmeroides*; †*Scombroclupea*; †*Enchodus*; †*Eurypholis*; †*Rhynchodercetis* or †*Belonostomus*; †*Ichthyotringa—*also referred to as †*Rhinellus*) from the Maastrichtian, and one chondrichthyan (*Galeus* or *Squalus*) and one teleost (*Clupea*) from the Paleocene (Koch & Nicolaus, 1969). Unfortunately, precise topographical information that could facilitate the rediscovery of most fossil-yielding localities and repository information for previously collected specimens was not provided in the original study.

The study of these fossil assemblages and their corresponding paleoenvironments offers unique opportunities for qualitatively evaluating the status of Tethyan marine pelagic ecosystems and biodiversity right before the K–Pg Extinction and possibly at its immediate aftermath, at the dawn of the Cenozoic. At the same time, it can help monitor the effects of the extinction event on open marine ecosystems and biodiversity in a paleogeographically constrained setting. In 2019 and 2020, we led exploratory missions in the vicinity of the town of Karpenisi, Eurytania, with the goal to discover and map fossiliferous horizons and localities and attempt their first systematic collection and study. Our efforts resulted in the discovery of several completely new localities yielding Maastrichtian teleosts, and the possible rediscovery of a previously reported Paleocene locality. We made a collection of over 50 specimens of body fossils of actinopterygians of varying levels of completeness from Maastrichtian, and a handful of similarly preserved elements from Paleocene exposures. This new fossil material and localities help ameliorate a major deficiency of the body-fossil record of actinopterygians from rocks surrounding the K–Pg Extinction. In this work, we report on fossils from the three most prolific localities of late Maastrichtian age and on one of Paleocene age. Furthermore, we discuss the significance and prospects of these fossil assemblages in the context of Maastrichtian–Paleogene transition in offshore marine environments.

### Geological setting and age of fossiliferous deposits

The area around the town of Karpenisi, Eurytania, continental Greece, is dominated by the pelagic sedimentary rocks of the Pindos Unit (also known as the “Pindos Belt” or the “Pindos Zone”; Koch & Nicolaus, 1969; Kallergis, Koch & Nicolaus, 1970; Fleury, 1980), which constitutes a tectonostratigraphic terrane that records ~160 Myr of Mesozoic offshore, and largely calcareous sedimentation on the passive Gondwanan margins of the so-called “Pindos Ocean”, which was in turn part of the greater Tethys Ocean during the Mesozoic and early Paleogene (Fleury, 1980; Papanikolaou, 2013). The Pindos Unit forms a NNW to SSE belt, which begins in Southern Albania, runs along western and central continental Greece and the Peloponnese, to reach Crete and Gavdos Island to the south and is nowadays arranged in the form of multiple thrust sheets (nappes) (Fleury, 1980; Papanikolaou, 2013). According to current tectonostratigraphic models, during the Mesozoic, the depositional basin of the Pindos Unit, along with the other terranes (“Units”) of the “external Hellenides” begun drifting towards the margin of the European continent, to which they accreted (Papanikolaou, 2013). The sedimentary rocks of the Pindos Unit in Eurytania entered the tectonic trench and began receiving clastic flysch-type deposits almost concurrently with the Maastrichtian–Danian boundary, although a certain degree of heterochrony in timing of onset of flysch deposition in the Pindos Unit is observed among different regions of continental Greece, Crete and Gavdos, (Koch & Nicolaus, 1969; Fleury, 1980; Papanikolaou, 2013). During the continental accretion and emergence processes associated with the Alpine Orogene, the Pindos Unit was displaced westwards, became subject to extensive tectonic deformation, and was thrusted over shallower water sedimentary facies contained within tectonostratigraphic terranes associated with the former Gondwanan continental margin (Fleury, 1980; Papanikolaou, 2013).

**Figure 1:**
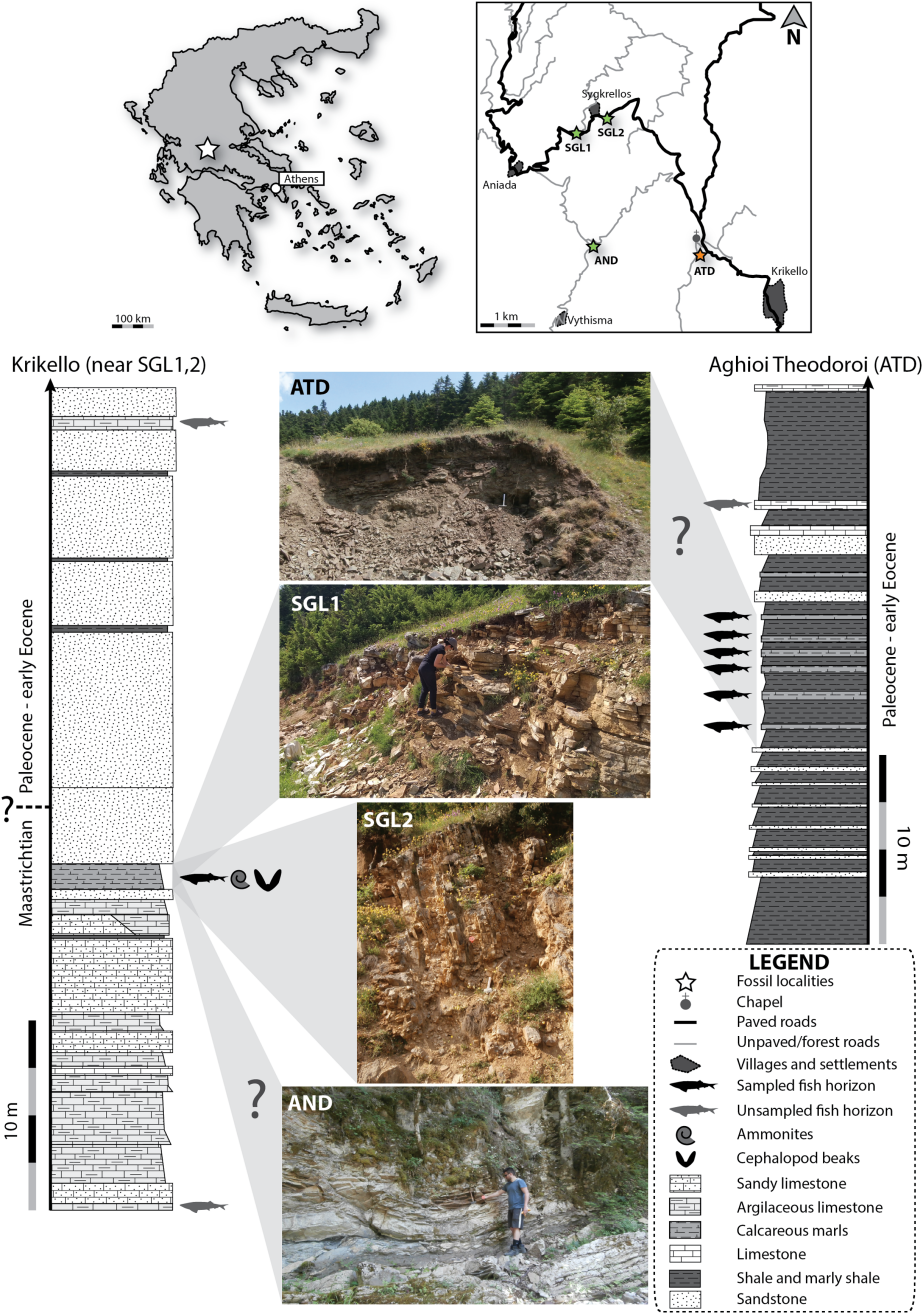
Locality and stratigraphic information. Stratigraphic logs and ages after (Koch & Nicolaus, 1969), and simplified. The placement of the K–Pg boundary is arbitrary, but shown to be contained within the flysch-related sandstones, following Fleury (1980). Localities AND and ATD are tentatively correlated with the corresponding horizons in the stratigraphic logs. Hammer in the pictures of SGL2 and ATD highlighted in white to give a sense of scale.

Upper Cretaceous lithologies of the Pindos Unit, in the vicinity of Karpenisi, Eurytania, are dominated by extensive successions of Turonian–late Maastrichtian pelagic platy limestones (“Plattenkalk”), with gradually diminishing siliceous lenticular inclusions, and varying terrigenous sandy or argillaceous inputs (Fig. 1; Koch & Nicolaus, 1969; Fleury, 1980). Distinctive thin layers of marly limestones, which overly the Plattenkalk, acting as harbingers of flysch sedimentation, can be recognized along the Pindos belt and are historically and informally referred to by geologists as the “transitional layers to the flysch” (Fleury, 1980). In Eurytania, these laminated marly layers contain fossils of ray-finned fishes, chondrichthyans, as well as cephalopod beaks and rare, poorly preserved ammonite shells (Koch & Nicolaus, 1969; Klug et al., 2020; this work). These transitional, marly facies in the vicinity of Karpenisi were dated to the late Maastrichtian–Danian, on the basis of the presence of the planktonic foraminifer †*Contusotruncana* (=†*Globotruncana*) *contusa* (Koch & Nicolaus, 1969), or, more precisely, to the late Maastrichtian (~70–67 Ma) on the basis of a more diverse planktonic foraminiferal sample, including: †*Contusotruncana* (=†*Globotruncana*) *contusa*; †*Globotruncana falsostuarti*; †*Globotruncana gansseri*; †*Globotruncanita* (=†*Globotruncana*) *stuarti* (Fleury, 1980; taxonomy and biostratigraphic ranges updated sensu Huber & Petrizzo, 2019). These fossiliferous transitional layers are also exposed in Gavdos Island, where they have been more recently dated to the middle–late Maastrichtian (Cavin, Alexopoulos & Piuz, 2012).

The exact placement of the K–Pg boundary has not yet been pinpointed in Eurytania, but the base of flysch-related sandy deposits was dated to the late Maastrichtian (Fleury, 1980). Overlying sandstone, marl and siltstone intercalations are dated as Paleocene—with the top-most layers possibly ranging into the early Eocene—on the basis of the complete absence of †globotruncanids, and the presence of Paleocene globigerinid Foraminifera (Koch & Nicolaus, 1969; Fleury, 1980). A thin limestone horizon containing fish fossils caps these intercalations and was dated to the early Eocene, on the basis of the foraminifers: †*Cuvillierina cayeuxi* (taxonomic validity is questionable); †*Discocyclina*; †*Nummulites*; †*Orbitoides*; †*Opertorbitoides* (possible synonym of †*Opertorbitolites*) (Kallergis, Koch & Nicolaus, 1970; taxonomy updated after Hayward et al., 2020). For the purpose of this work, we accept these biostratigraphic age estimates for the Maastrichtian and Paleocene fossiliferous horizons in Eurytania, but we stress the need to achieve better biostratigraphic resolution and obtain absolute ages—which are currently lacking—in the future. We note that the biostratigraphic age (Paleocene) of locality ATD is poorly resolved. Other than fossil fishes, plant remains, and amber were recognized in Paleocene rocks of the greater study area (Koch & Nicolaus, 1969). It should be noted that due to extensive tectonic deformation and heavy forestation it can be challenging to correlate fossiliferous horizons with different levels depicted in composite stratigraphic logs (Koch & Nicolaus, 1969; Kallergis, Koch & Nicolaus, 1970; Fleury, 1980).

### Fossil localities

#### Maastrichtian

We confidently correlate SGL1 (38°49′40.02″N; 21°48′16.71″E) and SGL2 (38°49′53.56″N; 21°48′37.91″E), which correspond to roadcut exposures flanking the village of Sygkrellos (Fig. 1), with the “transitional” marly limestone facies. We found †desmoceratid (?*Hauericeras* sp., ?*Kitchinites* sp.) and some indeterminate ammonites associated with fish fossils (Klug et al., 2020), which exclude a Danian age for the latter. Non-mineralized ammonite and coleoid jaws were also recovered from the same horizons (Klug et al., 2020). An additional locality, AND, is located on the banks of Aniada Creek, near the dirt road from Aniada to Vythisma (38°48′23.82″N; 21°48′32.12″E). The sampled horizon is tentatively correlated with the transitional, marly limestone horizon exposed in SGL1 and SGL2, and a late Maastrichtian age, on the basis of lithological similarities, but yielded no additional biostratigraphic markers.

#### Paleocene

Locality ATD [38°48′14.78″N; 21°50′2.61″E) lies well-within the area occupied by the Pindos flysch (Koch & Nicolaus, 1969; Kallergis, Koch & Nicolaus, 1970), and corresponds to a small quarry on the side of a dirt road, situated ~100 m from Aghioi Theodoroi Chapel (Fig. 1). Marly and clayey intercalations are exposed in this locality, but all specimens were found in debris at the base of the quarry, and we could not identify a certain horizon(s) as fossiliferous, due to low fossil concentration. Based on previous lithological and topographic descriptions, this locality was previously sampled by Koch & Nicolaus (1969), and can thus be placed with a certain degree of confidence in the Paleocene section of their stratigraphic log. Additional, previously reported fish-bearing Maastrichtian and Paleogene fossiliferous horizons and localities in the vicinity of Karpenisi have not yet been rediscovered. These seemingly include a calcareous fish-bearing horizon of Eocene age (Kallergis, Koch & Nicolaus, 1970; Koch & Nicolaus, 1969).

## Materials and Methods

Recovered fossil-bearing slabs were deposited and catalogued in the collections of the Museum of Geology and Paleontology of the National and Kapodistrian University of Athens (AMPG). Selected fossiliferous slabs were temporarily loaned to the Muséum national d’Histoire Naturelle, Paris (MNHN), for acid preparation and study. They were immersed in ~7% buffered formic acid, in order to achieve the dissolution of fossil covering matrix. Acid treatments lasted between 30 min to a maximum of two hours, and were followed by immersion in running water, for an equal, or longer amount of time. The exposed bone was protected using thin layers of paraloid glue. In this paper, we opted for historical anatomical terminology of actinopterygian cranial bones (Goody, 1969), for consistency with other works dealing with fossil teleosts. Throughout text and figures, extinct taxa are preceded by the dagger symbol (†).

## Systematic Paleontology

### Late Maastrichtian fossils

Actinopterygii sensu Goodrich, 1930

Teleostei Müller, 1845

Elopomorpha Greenwood et al., 1966

Elopomorpha indet.

Figures 2A–2D

**Figure 2:**
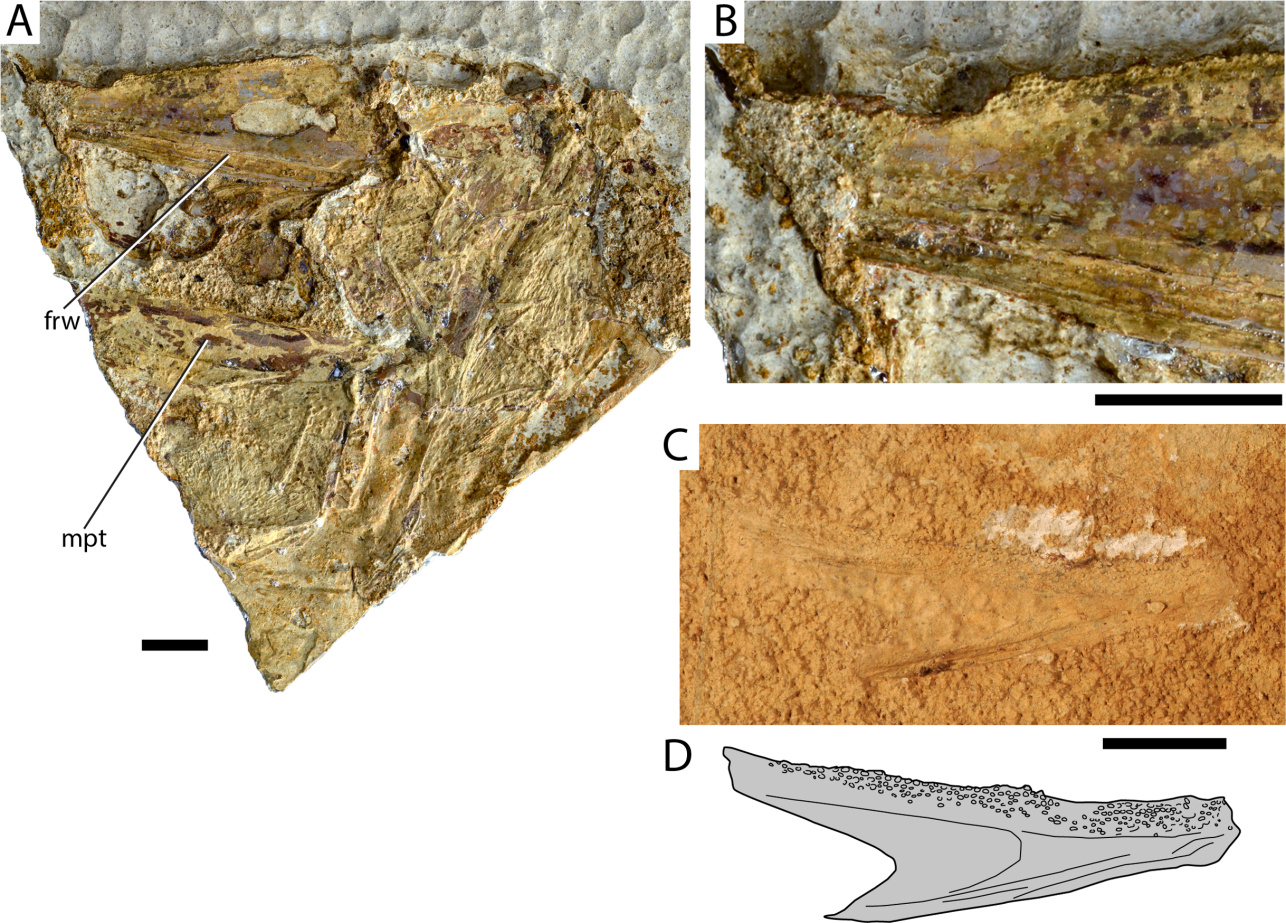
Maastrichtian Elopomorpha indet. (A) AMPG_SGL1_8 overview of specimen; (B) Magnified lateral view of region of dentary immediately behind the symphysis in (A), showing the arrangement of tooth alveoli; (C) AMPG_SGL1_25a, left dentary in medial view. (D) Interpretative drawing of (C). Abbreviations: frw, open sensory furrow on dentary; mpt, metapterygoid. Original photographs for (A) and (B) taken by L. Cazes. Scale bars equal 1 cm.

**Material.** AMPG_SGL1_8, partial skull with attached mandible; AMPG_SGL1_25a,b, isolated dentary

**Description.** The skull of AMPG_SGL1_8 is slightly disarticulated, with most skeletal elements being too incompletely preserved to be recognized. Much of the specimen comprises dermal bones ornamented with radiating series of tubercles, but the mode of preservation hampers the secure recognition and description of individual elements. An elongate and wide splint of unornamented, possibly endochondral, bone in the lower half of Fig. 2A possibly corresponds to a partial endopterygoid. One of the mandibles is preserved and is rotated in a way that the lateral side of the dentary is visible. The dentary is longer than high and tapers gently anteriorly. An open groove for the mandibular sensory canal is seen along its ventral margin. The region of the coronoid process is not well preserved but judging from the dorsal margin of the dentary it was likely gently produced. The occlusal surface of the dentary is very thin and bears numerous small tooth alveoli. The tooth patch seems to descend medioventrally. Anteriorly, towards the symphysis, the tooth patch widens and expands ventrally on the lateral surface of the bone. No ornamentation is visible on the dentary.

An additional isolated dentary (AMPG_SGL1_25a,b; Figs. 2C and 2D) exhibits a similar morphology and dense distribution of dental foramina with the mandible described above. The medial surface of the dentary is exposed. The posterior margin of the dentary forms a U-shaped indentation, with its dorsal arm expanding further posteriorly than its ventral arm. The dentary is gently constricted near the symphysis and displays a wide occlusal tooth patch posterior to the region of the symphysis. Tiny villiform or tuberous teeth are associated with some of the sockets, with the largest teeth being located near the dorsal margin.

**Remarks.** We attribute this material to Elopomorpha on the basis of several anatomical features. The most important pertains to the presence of an open furrow for the passage of the mandibular sensory canal along the ventrolateral margin of the dentary, which is one of the key synapomorphies of modern albuliforms, and several fossil forms historically treated as such, excluding †*Osmeroides* (Forey, 1973; Blum, 1991; Forey et al., 1996, 2003; Forey & Maisey, 2010). An open mandibular canal furrow can also be found in for example, the short and deep dentaries of clupeomorphs (Grande, 1985; Forey & Maisey, 2010). The tubercular ornamentation of dermal bones and the presence of tooth patches bearing numerous small foramina for villiform or tuberous teeth are consistent with an elopomoph attribution (Forey, 1973; Forey & Maisey, 2010). Tooth alveoli wrapping around the symphysis and expanding on the lateral surface of the dentary have been reported, together with the presence of an open mandibular canal, in the short-snouted †*Brannerion* from the Albian of Brazil (Blum, 1991; Forey & Maisey, 2010). Given the length of identified jaw bones, the Greek fish was probably long-snouted like for example, the Campanian elopomorph †*Istieus* (Siegfried, 1954; Forey, 1973) and unlike †*Brannerion* (Blum, 1991; Forey & Maisey, 2010). We note, however, that the systematic placement of Cretaceous elopomorphs relative to modern lineages is controversial (summarized in Dornburg, Friedman & Near (2015)). Elopomorphs have been previously reported in the Cretaceous of Eurytania, and were attributed to the genera †*Holcolepis* and †*Osmeroides* (Koch & Nicolaus, 1969). These two genera have since been shown to be synonymous, with †*Osmeroides* taking precedence over †*Holcolepis*. However, many species formerly included in †*Osmeroides* or †*Holcolepis* (e.g., the Cenomanian–Turonian putative elopid †*Davichthys lacostei* from Jebel Tselfat (Arambourg, 1954) have since been placed in different genera (Forey, 1973). The absence of any anatomical information on previously collected specimens (Koch & Nicolaus, 1969) and poor preservation preclude further systematic comments on the Eurytanian material.

Eurypterygia sensu Betancur-R et al., 2017

Aulopiformes Rosen, 1973

†Dercetidae Pictet, 1850

†Dercetidae indet.

Figures 3A and 3B

**Figure 3:**
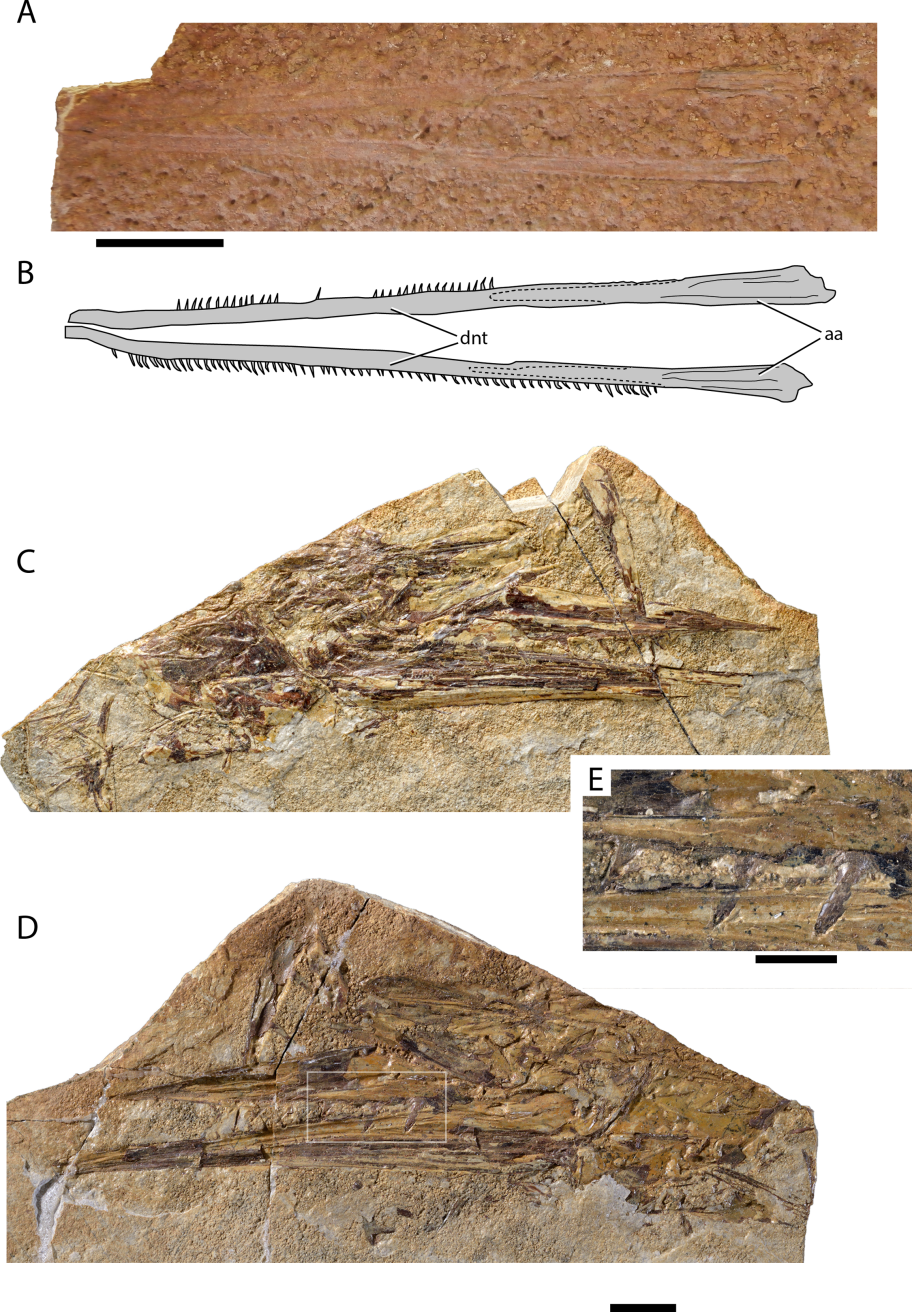
Maastrichtian longirostrine Aulopiformes. (A) †Dercetidae indet. lower jaw AMPG_SGL1_45; (B) interpretative drawing of (A). †Ichtyotringoidei gen. et sp. nov. AMPG_SGL1_14a,b. (C) Part, (D) counterpart. (E) Magnification of the region contained within the white box in (C), showing the anteriorly inclined fangs on the palatine bone. Original photographs for (C)–(E) taken by L. Cazes. Abbreviations: aa, anguloarticular; dnt, dentary. Scale bars for (A)–(D) equal 1 cm. Scale bar for (E) equals 5 mm.

**Material.** AMPG_SGL1_45, lower jaw

**Description.** Both long and slender hemimandibles are preserved on a weathered slab. The boundary between the dentary and the anguloarticular is faint, but there is no well-developed coronoid process. As evidenced by tooth imprints on the surrounding matrix, the occlusal surface of the dentary is occupied by a single row of closely spaced tiny conical teeth, most of which are weakly inclined posteriorly. The teeth reach the symphysis, the tip of which is broken off.

**Remarks.** The lack of a coronoid process in combination with the presence of multiple tiny teeth on this elongate jaw are characteristic for †dercetids (Goody, 1969; Da Silva & Gallo, 2011). Lower jaws previously attributed to either †*Rhynchodercetis* (†Dercetidae), or—erroneously—to †*Belonostomus* (†Aspidorhynchidae) have been figured by Koch & Nicolaus (1969). Our findings confirm the presence of †dercetids in the area, but a generic attribution cannot be attempted due to the incompleteness of the material at hand.

†Ichthyotringoidei Goody, 1969

†Ichthyotringidae Jordan, 1905

†Ichthyotringidae indet.

Figures 3C–3E

**Material.** AMPG_SGL1_1a,b partial skull and jaws; AMPG_SGL1_11, AMPG_SGL1_13 lower jaws; AMPG_SGL1_14a,b dermal palates and mandibles; AMPG_SGL1_23a,b anterior skull and hemimandible; AMPG_SGL1_26a,b, AMPG_SGL1_27 lower jaws; AMPG_SGL1_41a,b lower jaw; AMPG_SGL1_42a,b rostral fragments; AMPG_SGL1_53a,b,c, AMPG_SGL1_54a,b skull fragments.

**Description.** This probable new species is represented by numerous skull and jaw elements, often found disarticulated. We only provide a short description here, as this material merits more detailed anatomical and taxonomical examinations, which lie beyond the broader scope of this paper. The skull is shallow and elongate, with the preorbital region forming a prominent, acute rostrum. The tip of the rostrum is formed by a median element, which we interpret as a fusion of the two premaxillae. It is unclear whether this element supports fangs. The dorsal and lateral aspect of the rostrum is formed by the elongate dermethmoid and possibly the tightly incorporated maxillae. The toothed rim of the upper jaw is formed exclusively by a shallow and elongate palatine anteriorly and a shallow ectopterygoid posteriorly. The palatine bears large, anteriorly inclined fangs (Fig. 3E), flanked by a series of small conical teeth. The frontals are long but don’t reach far into the rostrum. They form a prominent but gentle constriction at the level of the orbits. A partial opercular series is only preserved in AMPG_SGL1_1a,b, and exhibits an opercle with a slightly posteriorly inclined anterior margin and a pointed, strongly convex posterior margin. A horizontal bony strut reinforces the opercle, reaching its posterior tip. The skull roof is largely unornamented. The opercle and subopercle exhibit few irregularly situated pits.

The mandibles are markedly elongate conforming to the overall shape of the rostrum. The dentary is the largest bone of the lower jaw and bears multiple anteriorly inclined fangs, with a series of smaller teeth between them being evident only in AMPG_SGL1_1a, b, which is also of larger size. Densely packed conical teeth occupy the posterior portion of the occlusal surface of the dentary. A short, blunt coronoid process is formed by the articular. The mandibular articulation is situated ventrally at the posterior surface of the mandible.

**Remarks.** We refer this material to †Ichthyotringidae indet. based on observed morphological similarities with species of the genus †*Ichthyotringa* (Amalfitano et al., 2020; Arambourg, 1954; Goody, 1969; Taverne, 2005), which pertain to the: (i) elongate and shallow skull forming a well-developed and acute prenarial rostrum; (ii) unornamented skull roof; (iii) presence of opercular strut; (iv) palatine and ectopterygoid being the main tooth bearing bones of the upper portion of jaw gape. We note however that the Eurytanian taxon differs from all known species of †*Ichthyotringa* in bearing multiple anteriorly inclined fangs, and also in exhibiting a coronoid process of the anguloarticular. Longirostrine cranial geometry and pronounced upper and lower jaw heterodonty differentiate the eurytanian fossil from other putative, shorter-snouted †ichthyotringoids, including the Turonian–Maastrichtian †*Apateodus* (see Da Silva & Gallo, 2011; Friedman, 2012; Goody, 1969; Newbrey & Konishi, 2015; but see Vernygora et al., 2018, for a recent agnostic phylogenetic placement of the genus) and the Albian †*Ursichthys* (Newbrey & Konishi, 2015). A more detailed taxonomic comparison and investigation of this material is warranted and will be attempted elsewhere. The presence of †*Ichthyotringa* in the Maastrichtian of Eurytania has been previously indicated by Koch & Nicolaus (1969), who also referred to it in the same text with its junior synonym †*Rhinellus*. We note that prior to our reassessment of the Eurytanian Maastrichtian fossils, †Ichthyotringidae were widely thought to be restricted to Cenomanian–Campanian deposits, thus, not reaching the K–Pg boundary (Goody, 1969; Dietze, 2009; Davis & Fielitz, 2010; Da Silva & Gallo, 2011; Newbrey & Konishi, 2015). Whereas the †ichthyotringoid †*Apateodus* was thought to have gone extinct in the early Maastrichtian (Friedman, 2012).

†Enchodontidae Woodward, 1901

†Enchodontidae indet. 1

Figures 4A–4C

**Figure 4:**
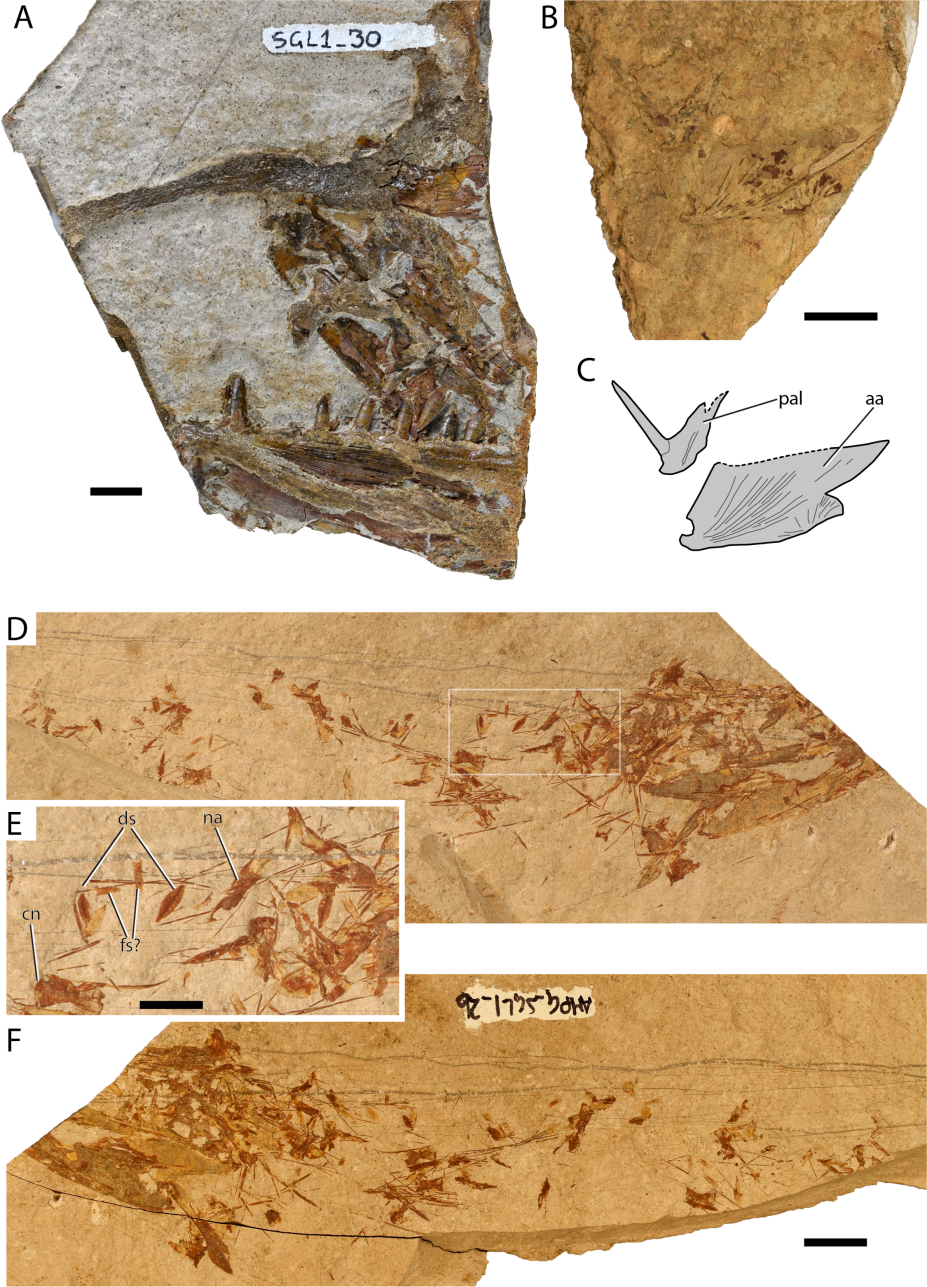
Maastrichtian †Enchodontidae. †Enchodontidae indet 1: (A) AMPG_SGL1_30 Anterior portion of skull preserving upper, lower jaws, and palatal ossifications; (B) AMPG_SGL1_33 weathered anguloarticular and palatine ossifications; (C) Interpretative drawing of anatomical structures in (B); †Enchodontidae indet 2 (AMPG_SGL1_2a,b): (D) overview of part; (E) magnification of the region contained within a white box in (D); (F) overview of counterpart. Abbreviations: aa, anguloarticular; cn, vertebral centrum; ds, almond-shaped dorsal scutes; fs?, putative flank scales; na, neural arches; pal, dermopalatine. Original photograph for (A) taken by L. Cazes. Scale bars for (A)–(D) and (F) equal 1 cm. Scale bar for (E) equals 5 mm.

**Material.** AMPG_SGL1_20, partial skull including mandibular and palatal elements; AMPG_SGL1_29a,b fragmentary palate and suspensorium and lower jaws; AMPG_SGL1_30, anterior portion of skull including upper and lower jaws; AMPG_SGL1_33, slab bearing anguloarticular and palatine bones; AMPG_SGL2_1, jaw fragment.

**Description.** Due to incomplete preservation, only the jaws and palate of AMPG_SGL1_30 can be described in sufficient detail. Other specimens are described when they can provide additional anatomical information. The medial side of the right premaxilla of AMPG_SGL1_30 is well-preserved. The occlusal surface of the premaxilla bears small conical teeth that decrease in size posteriorly. The maxillary teeth are partially overlain by a lateral, premaxillary lamina. Anteriorly, the premaxilla is weakly curved dorsally. Two thin, dorsally-posterodorsally running processes issue from the anterodorsal part of the bone. In life, these must have surrounded the tip of the anterior fang of the dentary when the jaw was closed. Both palatines are preserved, with the left one being slightly obscured by the left premaxilla, and the right one missing the distal end of its fang. The anteromedial side of the palatine bears a faint groove for the insertion of the ethmoid bone. The bone around the base of the palatine fang is somewhat thickened. The palatine fang at the anteroventral portion of the palatine is slightly curved, bears no striations, and must have been inclined posteriorly. The anterior portion of the right ectopterygoid is better preserved than the left one. This portion of the ectopterygoid bears three teeth, which are slightly better developed than their counterparts on the dentary. The largest of all preserved ectopterygoid teeth is more than half the length of the palatine fang. A posterodorsally pointing tooth overlapping the first tooth of the ectopterygoid was either taphonomically dislocated or corresponds to a replacement tooth. Additional ectopterygoid teeth must have been present posteriorly.

The left dentary forms a prominent anteroventral process. Laterally, it is ornamented with rostrocaudally running striations, and also forms a similarly directed trough, which begins posterior to the level of the anterior dentary fang. The ventral margin of the bone lacks ornamentation. Approximately three small conical teeth are situated anterior to the main dentary fang. The main dentary fang is straight, blade-like, and is situated immediately posterior to the level of the bone thickening. It is slightly longer than the longest preserved dentary tooth, but slightly shorter than the palatine fangs. There is a larger gap between the main fang and the following tooth, than between more posterior teeth. Smaller, possibly replacement tooth crowns, pointing posterodorsally, emerge from the base of dentary teeth. None of the teeth observed so far exhibits a posterior barb. Excluding the main fangs, large teeth on the ectopterygoid and dentary can have a weakly sigmoid anterior cutting edge. Teeth of the lower jaw and ectopterygoid are laterally compressed and form anterior and posterior cutting edges.

AMPG_SGL1_20 exhibits little in terms of diagnostic elements, apart from a toothed ectopterygoid. The latter bone is shallow, and exhibits at least six caniniform teeth. The second tooth from anterior seems to be slightly larger than the ones surrounding it. Immediately anterior to the ectopterygoid, there is a thickened dermopalatine, and traces of a single straight fang.

AMPG_SGL1_33, although much weathered, exhibits an enlarged palatine bone, which bears a single, uncurved, unbarbed fang. The anguloarticular forms a moderately developed coronoid process, a posteroventrally situated glenoid fossa, and is ornamented with pronounced striae.

**Remarks.** The presence of a single palatine fang in AMPG_SGL1_20 and AMPG_SGL1_33 is a synapomorphy of †Enchodontidae, supporting the inclusion of this material to this family. Only two other taxa, which can be easily excluded as likely candidates exhibit this feature. One is the †dercetid †*Ophidercetis*, which exhibits a long, acute rostrum (Taverne, 2005). The other is the Cenomanian †*Rharbichthys*, recently excluded from †enchodontids (Da Silva & Gallo, 2011; Díaz-Cruz, Alvarado-Ortega & Carbot-Chanona, 2019), in which the palatine bone expands far anterior to the base of the fang (Arambourg, 1954; Fielitz, 2004; Amalfitano et al., 2020). The early Late Cretaceous †enchodontid †*Eurypholis* and the more widespread †*Enchodus* were preliminarily identified in the Maastrichtian of the Pindos Unit, Eurytania (Koch & Nicolaus, 1969). Although the dentition alone can hardly separate †*Enchodus* from †*Eurypholis* (Forey et al., 2003), our findings clearly differ from †*Eurypholis* in exhibiting a marked anteroventral symphyseal process of the dentary, which is otherwise shallow in †*Eurypholis* (Goody, 1969; Forey et al., 2003). As a further indication of difference, the tubercular ornamentation of jaw bones is pronounced in †*Eurypholis* (Goody, 1969), though the ornamentation can prove less diagnostic as it is dependent on ontogenetic stage (Cavin, Alexopoulos & Piuz, 2012). The dentition plays an important role in distinguishing species of †*Enchodus*, and, despite being incomplete, SVT_30 allows for a discussion on potential diagnostic characters, and comparison with other penecontemporaneous species. SVT_30 exhibits more closely spaced dentary teeth than the †*Enchodus*-like skull previously figured (but not described) from the Maastrichtian of Eurytania (Koch & Nicolaus, 1969), though both specimens seem to lack barbed teeth. A partial skull of †*Enchodus* cf. *dirus*, exhibiting barbed teeth, was found in late Maastrichtian deposits of the Pindos Unit, in Gavdos Island (Cavin, Alexopoulos & Piuz, 2012). The above suggests the presence of more than one species of †*Enchodus* in the Maastrichtian of the Pindos Unit. Multiple †*Enchodus* species are known to have coexisted in other localities (e.g., Maastrichtian of Morocco, Arambourg, 1952). It is worth noting that the genus †*Enchodus* is considered paraphyletic (Díaz-Cruz, Alvarado-Ortega & Carbot-Chanona, 2019) and is in need of revision.

†Enchodontidae indet. 2

Figures 4D–4F

**Material** AMPG_SGL1_2a–c, partially disarticulated skeleton

**Description** The single specimen recovered is in a poor state of preservation. The anterior portion of the cranium is missing, whereas much of the trunk has been taphonomically lost and is largely disarticulated. Still, the overall shallow and elongate body shape of this animal can be deduced, based on the distribution of bones on the slab, as well as the elongate and depressed morphology of abdominal vertebrae and their neural arches and spines. Most cranial and anterior postcranial ossifications are hard to identify, as the anterior portion of the trunk and the pectoral girdle have been taphonomically telescoped anteriorly, disarticulating the skull. The skull appears to have been elongate and rather shallow, as evidenced by the long and narrow skull-roof bones, and elongate lower jaws. The skull roof exhibits a slender and elongate pterotic, bearing the open supraorbital sensory canal. Another dermal element is preserved lying along the posterior part of the medial margin of the pterotic, bears traces of the mediolaterally directed, open supratemporal sensory canal, and is, thus, identified as the parietal. A disarticulated broad, shield-like element lies posterodorsal to the parietal, this being either an enlarged supraoccipital, or the other parietal. The cranial roof bones are ornamented with a dense network of pits and ridges. The posterior portions of both lower jaws are preserved. The articulation facet is situated on the posterior tip of the anguloarticular. Anterior to the articular facet, the anguloarticular gains height gently to form a rather shallow coronoid process. On the lateral surface of the bone, striae radiate anteriorly from the articular facet.

The first few abdominal vertebrae are stocky, and almost as long as high, exhibiting a gentle mid-length constriction. However, immediately posterior to the last cranial element, the abdominal vertebrae become elongate hourglass-shaped, displaying a conspicuous mid-length constriction. The neural arches are autogenous and are mostly found disarticulated in our specimen. Their posterior tip projects further than the centrum, forming robust postzygapophyses. The slender neural spine is located at the posterodorsal corner of the neural arch. No traces of lateral bony struts, or strong transverse processes are observed on the centra. Remnants of a median fin are preserved in the abdominal region, but pterygiophores or pelvic plates are absent, and we cannot determine whether this is an in-situ pelvic fin, or a disarticulated dorsal fin.

At least two types of scales are observed. The first type, which we identify as belonging to the mid-dorsal series, is narrow and almond shaped, with acute anterior and posterior tips, which are in turn connected by a mid-line ridge. Pits and ridges radiate from the center of each scale. The second type probably extended along the dorsal portion of the flank, but only few of these broadly quadrangular elements are preserved. Some scales that approach triangular shape are observed in the posterior abdominal-anterior caudal region, but these are not preserved well enough to be safely assessed.

**Remarks** The almond-shaped mid-dorsal scutes—if identified correctly—and the presence of an open supratemporal sensory canal support a placement of this fossil within †enchodontids (Da Silva & Gallo, 2011; Vernygora et al., 2018), though this attribution is tentative. AMPG_SGL1_2a–c exhbitis little overlap of preserved elements with the previously described †enchodontid, but we tentatively describe it here separately. It can also be easily differentiated from the †ichthyotringid found in the same locality on the basis of its lower jaw shape and ornamentation, which exhibits a shorter but wider coronoid process, and pronounced striated ornamentation. The honeycomb (pits and ridges) ornamentation pattern of skull roof bones was also not observed in any of the †ichthyotringid specimens recovered. Furthermore, in †ichthyotringids neural arches are fused to their corresponding centra (Goody, 1969; Taverne, 2006). The squamation pattern and shape of individual elements (e.g., presence of ovoid to rhomboidal mid-dorsal scales and deep flank scales) sets this form immediately apart from most of the slender and elongate bodied †dercetids, which bear characteristic triradiate, or triangular and slender flank scales; or completely lack scales (Siegfried, 1954; Goody, 1969; Chalifa, 1989; Da Silva & Gallo, 2011; Friedman, 2012; Vernygora et al., 2018). This fossil is readily distinguishable from the long-snouted †*Apateopholis* (Goody, 1969; Forey et al., 2003), as its lower jaw articulation is situated without doubt posterior to the level of the orbit.

The combination of somewhat quadrangular flank scales and ovoid–almond-shaped mid-dorsal scutes broadly matches that of †eurypholins (Goody, 1969; Forey et al., 2003; Fielitz, 2004) and specifically that of the long-bodied †*Saurorhamphus* (Chalifa, 1985; Alvarado-Ortega, Ovalles-Damián & Blanco-Piñón, 2009). The long-bodied †*Palaeolycus* (von der Marck, 1885; Siegfried, 1954) is another possible candidate, as it exhibits numerous mid-dorsal scutes, but lacks any lateral scute rows. Phylogenetic affinity with the aforementioned taxa would require more complete material to test. Narrow oval to almond-shaped mid-dorsal scutes are also present in the Cenomanian †*Unicachichthys* (Díaz-Cruz, Alvarado-Ortega & Carbot-Chanona, 2016), but the overall body shape of the latter is deeper than that of the Greek fossil. The conceived mid-dorsal scales are much narrower than those seen in †*Eurypholis*, †*Enchodus*, †*Dagon* (Pictet, 1850; Goody, 1969; Díaz-Cruz, Alvarado-Ortega & Carbot-Chanona, 2019). Yet, the Greek fossil in question bears pits-and-ridges cranial roof ornamentation indicating that it belongs to a different genus. †*Eurypholis* has been previously listed as present in the Maastrichtian of Eurytania (Koch & Nicolaus, 1969), but no data were presented to support its presence. It is thus possible that previous authors encountered the same taxon described herein and linked its squamation pattern to †*Eurypholis*. We note some ornamental similarities between the anguloarticular of AMPG_SGL1_33 and the poorly preserved anguloarticulars of AMPG_SGL1_2a–c (Figs. 4B and 4C).

Eurypterygia indet.

Figures 5A–5D

**Figure 5:**
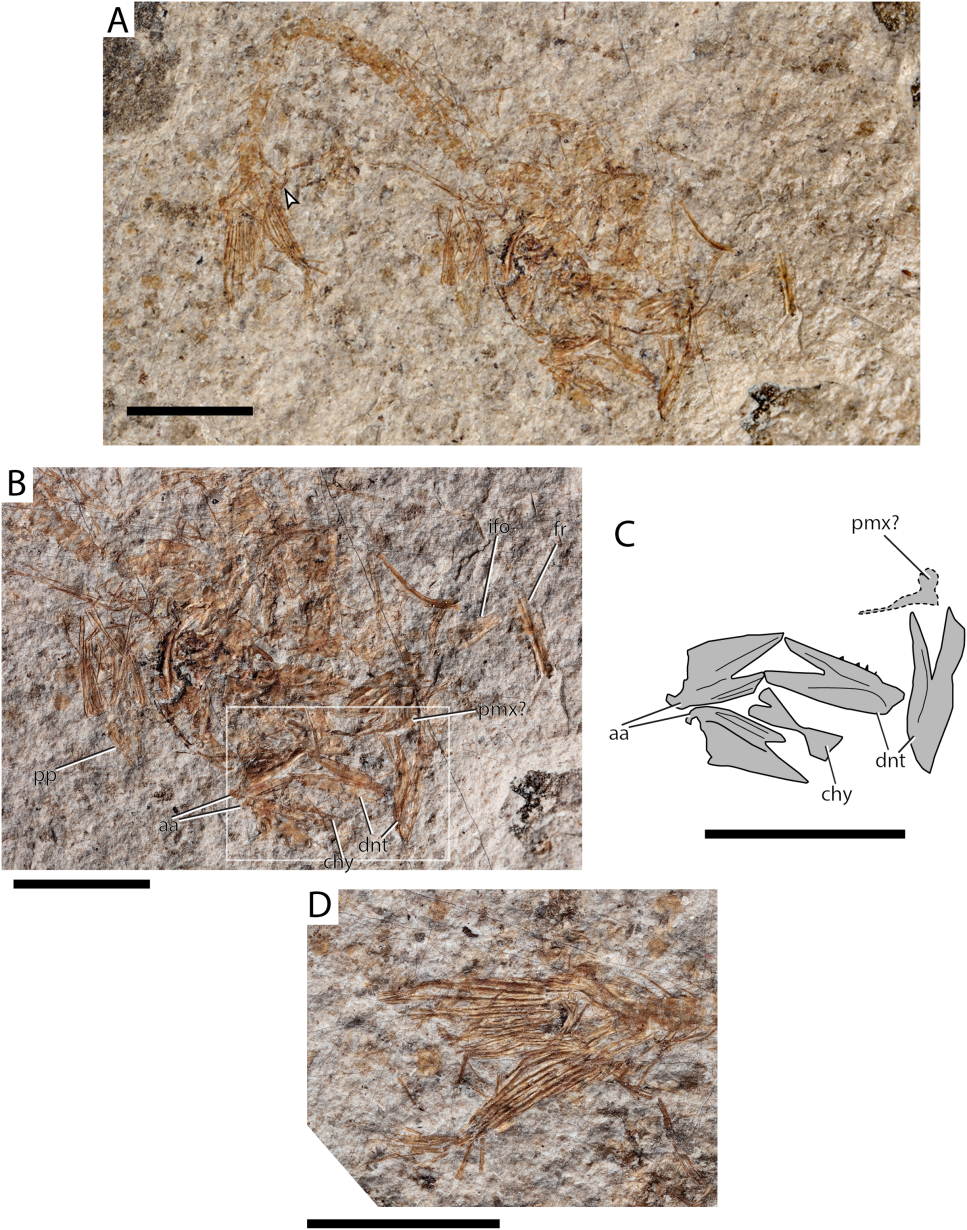
Maastrichtian Eurypterygia indet. (A) Complete specimen AMPG_AND_2b. Arrowhead indicates the posteriormost segment of the pectoral fin; (B) closeup of the disarticulated head; (C) magnified interpretative drawing of jaw elements contained within a white box in (B); (D) closeup of caudal fin. Abbreviations: aa, anguloarticular; chy, (anterior) ceratohyal; dnt, dentary; fr, frontals; ifo, infraorbital; pmx?, putative premaxilla; pp, pelvic plate. Original photographs for (A), (B) and (D) taken by L. Cazes. Scale bars equal 5 mm.

**Material.** AMPG_AND_2a,b, partially disarticulated individual.

**Description.** A tiny, possibly immature, individual (SL slightly over 30mm) is preserved in a curled position and partially disarticulated state as part and counterpart. Several anatomical structures can be described. The frontals are narrow and elongate and form a gentle curve at the level of the orbit. Their anterior margin is pointed, while their posterior margin is almost straight. The suture between the two frontals is straight. Traces of quadrangular parietals are preserved, exhibiting a post-frontal widening of the skull roof. A disalrticulated broad quadrangular canal-bearing bone lies between the dislocated skull-roof and the rest of the specimens and is most likely one of the anteriormost elements of the infraorbital series. A possible preopercle seems to bear an anteriorly directed ventral arm. We only tentatively identify a badly preserved element as a premaxilla with a robust ascending process. The lower jaw is much stockier than that observed in other fishes from the area. The anguloarticular forms a triangular coronoid process, and its articulation with the quadrate is wide, situated posteriorly and points dorsally. The dentary is almost 2,5 times as long as high, rather straight, and blunt anteriorly. Its posterior margin forms a deep V-like indentation. Traces of tiny teeth are observed on its occlusal margin. The anterior ceratohyal is strongly constricted at about mid-length.

Aspects of the postcranial skeleton are also visible. The centra are higher than long, and seem to bear slender neural and haemal spines, which issue from the anterior portion of the corresponding arch. Neural and haemal arches seem to be fused with their corresponding centrum. Interestingly, the pectoral fins are markedly elongate, almost reaching the caudal fin. The pectoral fin segments are longer than high. The pelvic girdle is situated at a thoracic, or jugular position. Although a post-mortem displacement cannot be excluded, the pelvic bones and fin rays are mostly in connection, which suggests that displacement was limited. The pelvic bones are pointed anteriorly. An exact count of the pelvic fin rays could not be established, but must have been around eight or nine. There is no pelvic-fin spine. The caudal fin is forked, and shows at least five procurrent spines. The endoskeleton of the caudal fin is not adequately preserved to allow for a more detailed description, but the tip of the vertebral column is upturned. Cycloid scales are scattered throughout the extent of the specimen.

**Remarks.** This material is not well-preserved enough to establish its precise phylogenetic position, but its peculiar combination of features suggests it differs from all other teleosts found in the locality. The position of the pelvic skeleton, in conjunction with the robust ascending process of the putative premaxilla, would support an attribution to acanthomorphs or a closely related group. However, the absence of fin spines (a well-defined—although not universal—feature of acanthomorphs) and the overall poor preservation of the material prevent us to go further than Eurypterygia indet. for this specimen. The pelvic fins and girdle tend to insert anteriorly in eurypterygians, a clade that includes aulopiforms, myctophiforms and acanthomorphs (Stiassny & Moore, 1992; Yamanoue, Setiamarga & Matsuura, 2010). Most Aulopiformes have pelvic fins in a sub-thoracic or abdominal position (i.e., they insert posteriorly to the pectoral girdle), with a few exceptions (e.g., alepisaurids, †enchodontids) (Goody, 1969). Myctophiformes revert to abdominal pelvic girdles. Conversely, pelvic fins and girdles have a thoracic or jugular position and insert anterior to the pectoral girdle in acanthomorphs (Stiassny & Moore, 1992; Yamanoue, Setiamarga & Matsuura, 2010; Davesne et al., 2016) and in †ctenothrissiforms, an enigmatic Late Cretaceous eurypterygian group (Goody, 1969). The morphology of the Eurytanian specimen suggests it was a relatively elongate animal, unlike most known Late Cretaceous acanthomorphs, that tend to be more deep-bodied. Elongate acanthomorphs with thoracic or jugular pelvic fins did exist in the Campanian-Maastrichtian though, for example, ophidiiforms and putative batrachoidiforms (Bannikov & Sorbini, 2000; Carnevale & Johnson, 2015).

Teleostei indet. morphotype 1

Figure 6A

**Figure 6:**
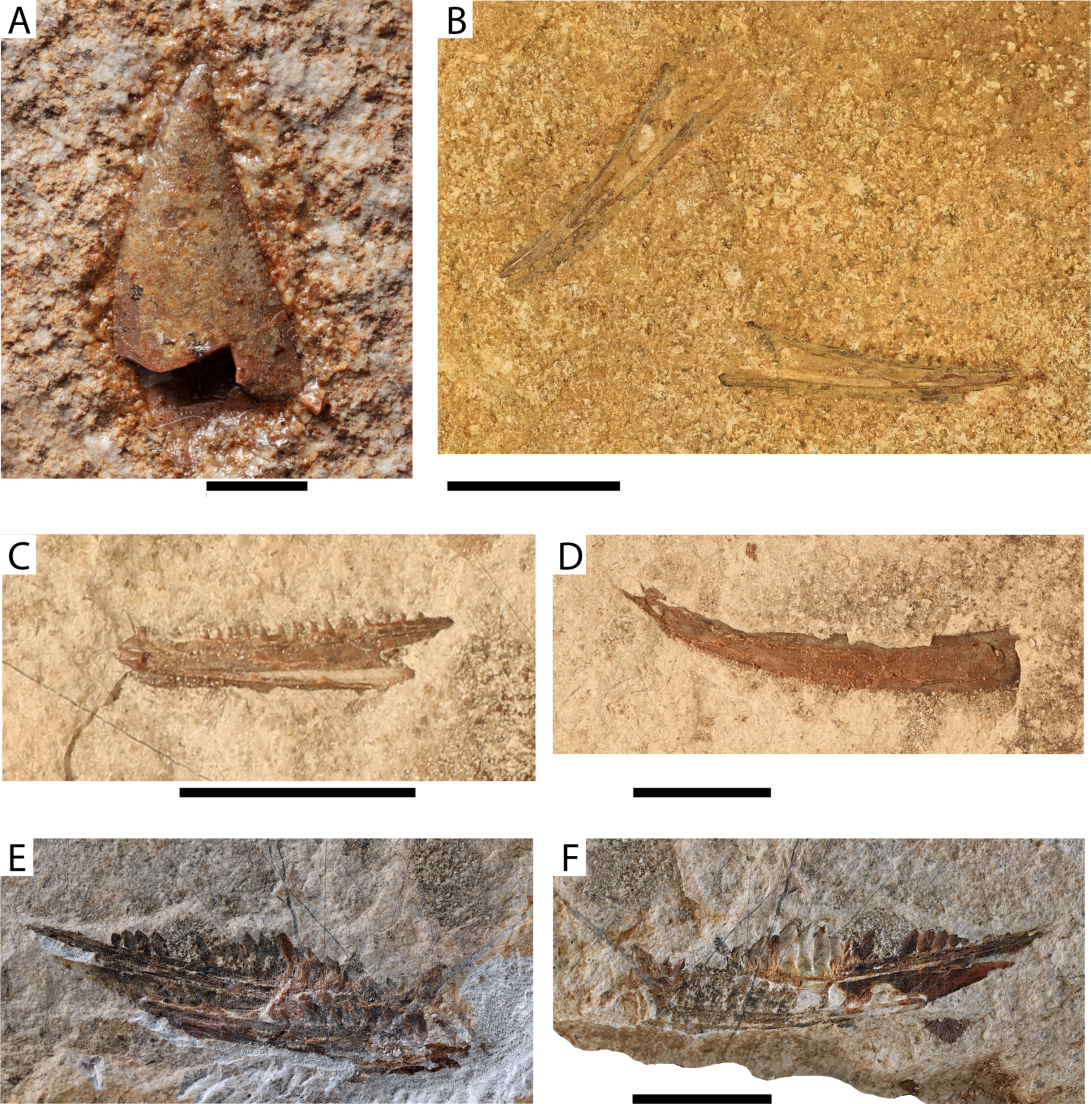
Maastrichtian unidentified teleost material. (A) Teleostei indet. morphotype 1 (AMPG_SGL1_5); Teleostei indet. morphotype 2: (B) dentaries from the same individual (AMPG_SGL1_32); (C) dentary (AMPG_SGL1_10b); (D) Teleostei indet. morphotype 3, maxilla (AMPG_AND_6); (E and F) Teleostei indet. Morphotype 4, dentigerous bone (AMPG_SGL1_15a,b) possibly belonging to Aulopiformes, part and counterpart. Original photographs for (A), (E) and (F) taken by L. Cazes. Scale bar for (A) equals 2 mm; Scale bars for (B)–(F) equal 1 cm.

**Material.** AMPG_SGL1_5, tooth crown

**Description.** The triangular crown forms an acute apex and is laterally compressed and slightly curved (conceivably) mesially. It forms distal and mesial cutting edges, both of which appear to reach the apex and bear small closely packed serrations. Faint striations are also visible near the base of the tooth. No differentiated acrodin cup is visible, but the apical enameloid is dense. The proximal part of the crown and the base are destroyed, leaving the hollow pulp cavity exposed. Apicobasal striae of enameloid are seen in the damaged internal proximal part of the tooth.

**Remarks.** This tooth is too incomplete to be identified at a lower taxonomic level, but derives from a vertebrate taxon different from the ones described below. An open pulp cavity is indicative of osteichthyan, rather than chondrichthyan affinities, although replacement teeth of elasmobranchs can also sometimes exhibit an open pulp cavity. Several teleosts from similarly aged deposits are known to bear comparably shaped serrated tooth crowns (e.g., †saurodontids, or the †ichthyotringid †*Apateodus*, see Friedman, 2012), but a detailed comparison would require better preserved material. The possibility that this fragmented tooth derives from a non-actinopterygian osteichthyan is negligible, given the pelagic marine setting it was found in.

Teleostei indet. morphotype 2

Figures 6B and 6C

**Material.** AMPG_SGL1_10a,b, slab with isolated dentary; AMPG_SGL1_32, slab with two dentaries

**Description.** The dentary is elongate and shallow, and mostly straight but gently curves dorsomedially towards its anterior tip. The symphyseal region is forms a slight ventral thickening. The posterior margin of the bone forms a deep U-like indentation for the insertion of the anguloarticular. The sensory canal is contained in an open furrow that trails the lateroventral surface of the bone. The occlusal margin of the bone bears a single row of small, anterolingually curved caniniform teeth, which in turn bear small acrodine caps.

**Remarks.** We did not identify any apomorphies to attempt a less inclusive attribution of this element, but the dentition does not match that of other recognized taxa. The elongate shape of the jaws and open mandibular sensory furrow suggest a possible attribution of these elements to some elopomorph taxon, different than the one described above. We note that Koch & Nicolaus (1969) reported two elopomorph fossil morphotypes from the area. This material might correspond to one of them.

Teleostei indet. morphotype 3

Figure 6D

**Material.** AMPG_AND_6, left maxilla

**Description.** The maxilla is elongate, gently curved, and rather short in height. Anteriorly, there are traces of a robust articular process. Posteriorly, the maxilla increases in height gradually. Numerous closely spaced, and ventrally directed villiform teeth, likely forming a single row, issue on the occlusal surface of the maxilla.

**Remarks.** Due to its thinness and gentle curvature, the seemingly robust articular process and the presence of a single row of ventrally directed teeth, the maxilla in question best resembles elopomorphs known from other late Cretaceous marine assemblages of the western Tethys (e.g., Jebel Tselfat, (Arambourg, 1954); Westphalian occurrences, (Siegfried, 1954); Cenomanian of Namoura, Lebanon (Forey et al., 2003), which can help tentatively constrain its taxonomic status. This maxilla might derive from the same elopomorph taxon as the skull material described above. However, similarly shaped maxillae are also encountered in actinopterygians outside elopomorphs, such as the Late Cretaceous stomiiform †*Paravincinguerria*, but in the case of the latter the teeth would be directed anteriorly (Carnevale & Rindone, 2011).

Teleostei indet. morphotype 4

Figures 6E and 6F

**Material.** AMPG_SGL1_15a,b, dentigerous bone; AMPG_SGL1_48, dentigerous bone

**Description.** The left and right counterparts of a dentigerous bone (AMPG_SGL1_15a,b) are compressed in a single plane, with one slightly overlapping with the other. Posteriorly, and between the two bony counterparts, there is evidence of another, smooth bony element, which forms an elongate and acute posterior tip. The yet unidentified elements in question are either partial dentaries, or dentigerous dermal palate bones, such as ectopterygoids. These bones form a thickened base, which in turn bears a densely packed row of posteriorly inclined teeth along the length of its occlusal surface. A shallow furrow extends along the length of the lateral(?) surface of the thickened base. The teeth are laterally compressed and blade-like, with their crowns forming anterior and posterior unserrated cutting edges. The cutting edges reach the acute apex. Thickened and faintly striated bony bases are evident in more anterior teeth, and form about a third of total tooth length. Anterior teeth are much longer than wide, but the dentition gradually reduces in height posteriorly. The teeth are closely spaced, with the posterior cutting edge of one tooth often overlapping with the anterior cutting edge of its immediately posterior tooth. A similar tooth pattern is observed in the less well-preserved AMPG_SGL1_48.

**Remarks** Collectively, the teeth seem to transform the enigmatic dentigerous bone into a slicing apparatus, conceivably specialized for soft-bodied prey. To our knowledge, no comparable elements have been previously described from any Cretaceous or Paleogene site. A recent collection of material from the late Maastrichtian of the Pindos Unit in Gavdos Island, Greece (T. Argyriou, 2019, personal observations; see Cavin, Alexopoulos & Piuz (2012) for more information on the locality), has yielded more diagnostic and better-preserved dentigerous bones from a single individual, characterized by similar dentition (to be treated elsewhere). We believe that these peculiar elements derive from a yet undescribed teleost genus and species, possibly belonging to Aulopiformes.

### Paleocene fossils

Clupeiformes Bleeker, 1859

Clupeoidei sensu Grande, 1985

Clupeidae Cuvier, 1817

Clupeidae indet.

Figures 7A–7E

**Figure 7:**
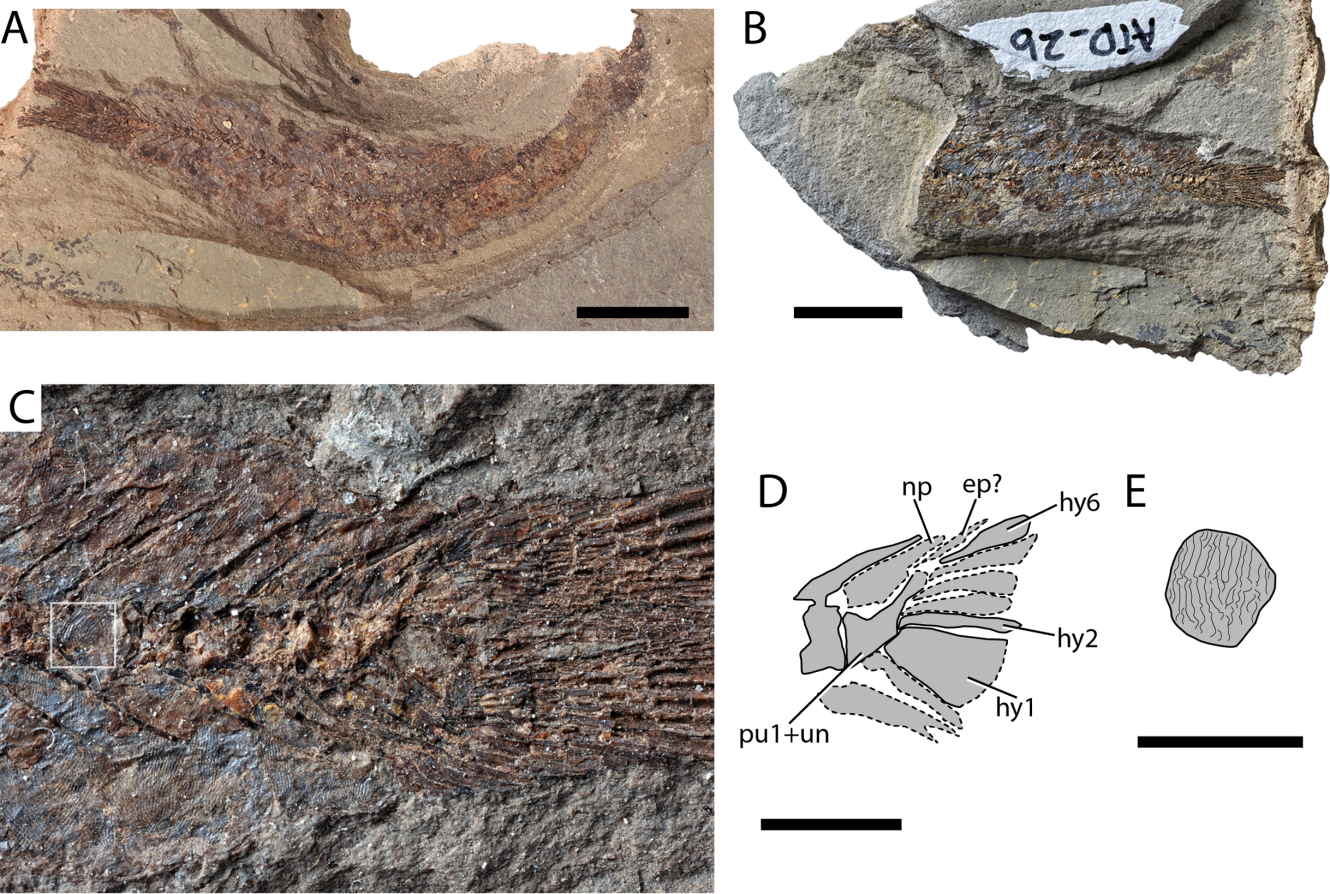
Paleogene (Paleocene?) Clupeidae indet. (A) Part (AMPG_ATD_2a).; (B) counterpart of tail (AMPG_ATD_2b); (C) magnified caudal region of (B); (D) interpretative drawing of caudal skeleton; (E) magnified interpretative drawing of scale contained in a white box in (C). Abbreviations: ep?, putative epural; hy, hypural; np, neural process; pu1+un, fused preural centrum and uroneural. Original photograph for (B) and (C) taken by L. Cazes. Scale bars for (A) and (B) equal 1 cm. Scale bar for (C) and (D) equals 2 mm. Scale bar for (E) equals 1 mm.

**Material.** AMPG_ATD_2a,b, part and counterpart of caudal region and fin

**Description.** The slabs split sagitally through the vertebral column. Preural centra are hourglass-shaped and slightly longer than high and bear thin dorsal and ventral prezygapophyses. Delicate neural and haemal arches and spines issue from about might length of each centrum. At least five dorsal and six ventral procurrent rays are respectively supported by the posterior neural and haemal arches. What we identify as the first preural centrum bears a broad epineural process and a fused uroneural. Approximately six hypurals are present, with the ventral-most (first hypural) being greatly expanded. The second hypural reaches further anteriorly, but remains unclear whether it fuses with the first ural centrum. The caudal fin is forked, and is composed by over 20 segmented rays, slightly more than half of which forming the dorsal lobe. Fin rays of the pelvic fin are visible, but only faint traces of a narrow anal fin. Median dorsal or abdominal scutes were probably absent. The body is covered in circular cycloid scales, segmented by a densely packed series of dorsoventrally running striae (Fig. 7E).

**Remarks.** The fusion of the first ural centrum with the second hypural—one of the main synapomorphies of clupeomorphs (Grande, 1985; Wiley & Johnson, 2010)—could not be assessed in the Greek specimen. However, we note similarities between the compound centrum of the Greek fossil to that of clupeoids, which results from the fusion of a uroneural with the first preural centrum, and one or two ural centra (Grande, 1985). The expanded neural process of the compound preural centrum best resembles that of for example, †*Trollichthys* and dussumieriins (Grande, 1985; Marramà & Carnevale, 2015a), and less so that of †*Bolcaichthys* (Marramà & Carnevale, 2015b). A clupeoid, and also clupeid, attribution is further supported by scale morphology. Scales exhibit vertically arranged, uninterrupted striae, such as those found in many clupeids, including †*Trollichthys* and †*Bolcaichthys* (Szymczyk, 1978; Grande, 1985; Marramà & Carnevale, 2015a, 2015b; Bräger & Moritz, 2016), but not in alosins (Szymczyk, 1978; Bräger & Moritz, 2016), or †*Eoalosa* (Marramà & Carnevale, 2018). The presence of clupeids tentatively attributed to the recent genus *Clupea*, in the Paleogene of Eurytania, has been previously reported by Koch & Nicolaus (1969, Pl. 32:4*)*. Clupeomorphs are known since the Early Cretaceous (De Figuereido, 2009). Aside from some fragmentary and questionably interpreted material from the Campanian–Maastrichtian of Nardò, Italy (Taverne, 2004; Taverne, 2007; see criticism in Marramà & Carnevale (2015a, 2018), the earliest known clupeids come from the Turonian (Alvarado-Ortega et al., 2020), followed by findings from the Paleocene of Mexico (Alvarado-Ortega et al., 2015). They became common components of Eocene and younger Tethyan ichthyofaunas (Grande, 1985; Jerzmańska, 1991; Marramà & Carnevale, 2015a, 2015b, 2018).

Stomiiformes sensu Harold & Weitzman, 1996

Stomiiformes indet.

Figures 8A–8D

**Figure 8:**
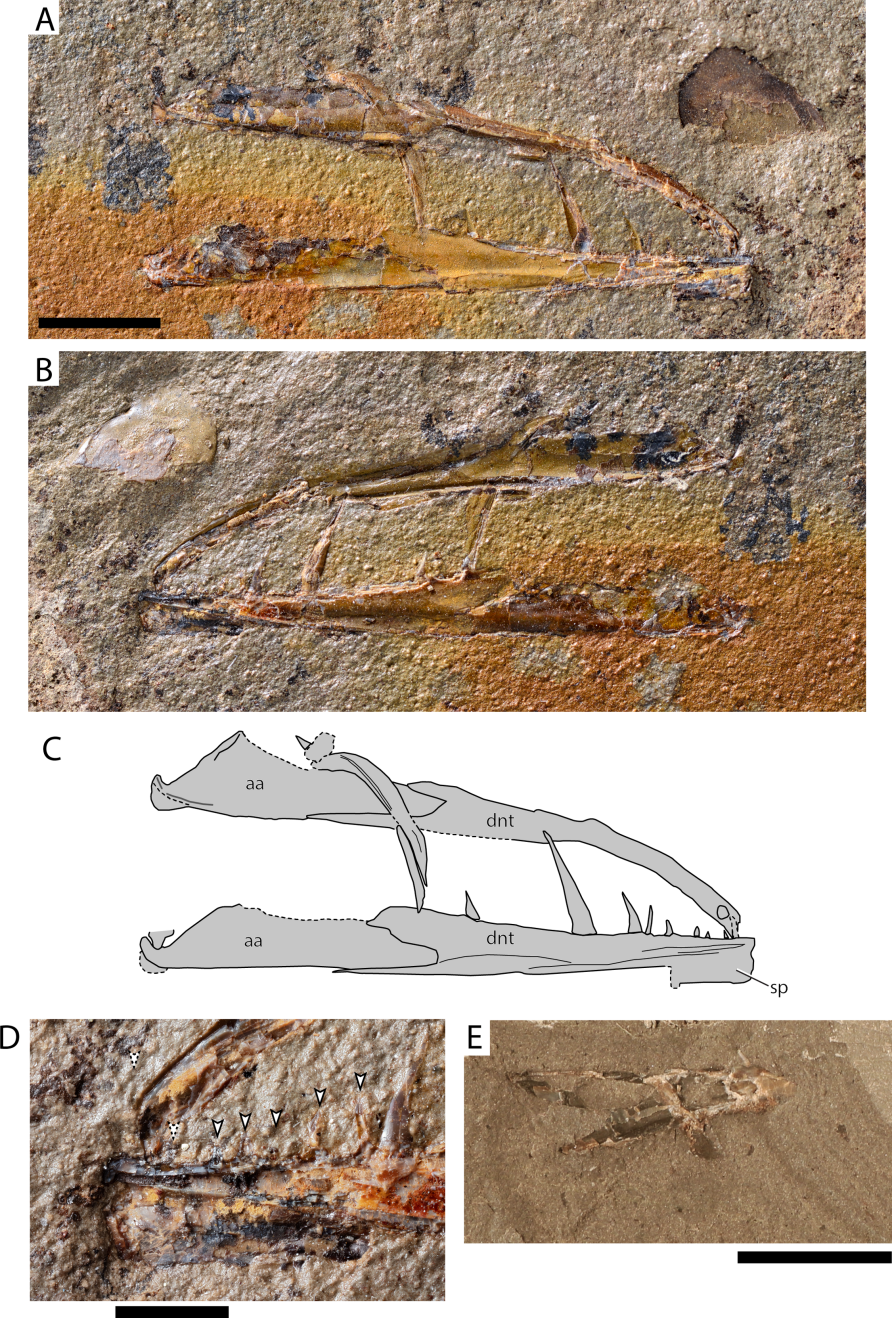
Paleogene (Paleocene?) Stomiiforms indet and Teleostei indet. Stomiiformes indet. AMPG_ATD_1a,b (A) part; (B) counterpart; (C) interpretative drawing of (A); (D) detail of the symphyseal region of (B). Arrowheads indicate near-symphyseal teeth, with the anterior two teeth being tentatively recognized. (E) Teleostei indet. jaw AMPG_ATD_3b. Abbreviations: aa, Anguloarticular; dnt, dentaty; sp, symphyseal process. Original photograph for (A), (B) and (D) taken by L. Cazes. Scale bars for (A)–(C) and (E) equal 1 cm, scale bar for (D) equals 2 mm.

**Material.** AMPG_ATD_1a,b, part and counterpart of left and right lower jaws

**Description.** The lower jaws are long, slender, with the dentaries gently arching medially. The anguloarticular is elongate, occupying over a third of the jaw, and shallow. The lower jaw articulation is situated at the terminal end of the anguloarticular and forms a wide, dorsally pointed U-like fossa. The posteroventral tip of the lower jaw, which possibly comprises the retroarticular, is rounded. Immediately anterior to the articulation facet, the anguloarticular forms a shallow coronoid process, the posterior margin of which forms a ~45° angle with the ventral margin of the bone. The posterior margin of the dentary is V-shaped, with its dorsal arm being higher but shorter than its skinny, elongate ventral arm. The dorsal margin of the dentary is thick, whereas the ventral is laminar. The dentary symphysis is particularly well-developed and expands ventrally, forming a sub-quadrangular plate. We observed no traces of sensory foramina on the dentary. At least 10–11 teeth of several lengths are born by the dentary in a single row. Four posteriorly inclined fangs are seen on the posterior three quarters of the dentary, with the two being much larger than the other two. The anteriormost fang is gently recurved. Six–seven small conical to recurved teeth, or traces of, are seen in the anterior fourth of the dentary, between the symphysis and the first large fang. The teeth have a circular outline in cross section, lack cutting edges and do not form posterior blades or barbs. The tooth enameloid is almost smooth, but microscopic bumps are seen in high magnifications. A weakly mineralized zone between tooth crown and bony base is observed in some teeth (Type 2 of Fink (1981)).

Fragments of putative upper jaw bones are also present in this specimen. A thin, edentulous curved bone crosses one of the jaws. Its length is approximately a quarter of that of the mandibles. It forms a thickened shelf along its length and its anterior tip curves dorsally. This element is either a supramaxilla, or an edentulous maxilla. A small quadrangular shard of bone and a tooth are seen on the anterior tip of the supramaxilla, which likely formed part of the premaxilla or maxilla, or a toothed pharyngobranchial (as in e.g., †*Primaevistomias*, Prokofiev & Bannikov, 2002; Prokofiev, 2005). A partial cycloid scale is seen above the tip of the jaw, but is unclear if it derives from the same individual.

**Remarks.** The low and elongate jaw, bearing fang-like recurved teeth, and forming a thin ventral lamina and a strong symphysis are similarities (but not synapomorphies) of the Greek fossil with certain Stomiiformes (Fink & Weitzman, 1982; Harold & Weitzman, 1996). Four “families”—including the paraphyletic but widely used “Phosichthyidae”—are recognized within Stomiiformes (Harold & Weitzman, 1996; Betancur-R et al., 2017). The dental pattern of the dentary of the Greek fossil, comprising a single row of teeth of various sizes, can help rule out sternoptychiids and “phosichthyiids”, which exhibit uniform dentition, and as a rule lack fangs (Weitzman, 1967; Weitzman, 1974; Nam, Ko & Nazarkin, 2019). Members of Gonostomatidae (e.g., *Gonostoma*) (Harold, 1998) and Stomiidae (e.g., *Photostomias*, or *Malacosteus*) (Fink, 1985) exhibit a heterodont dentition on the dentary comprising small teeth and fangs, which are arranged in a single row. We note some similarities in overall jaw morphology and the presence of small teeth preceding the fangs on the dentary between the eurytanian specimen and the early gonostomatid †*Primaevistomias* from the middle Eocene of Northern Caucasus, although the latter genus exhibits several small teeth between the large fangs of the dentary (Prokofiev & Bannikov, 2002; Prokofiev, 2005). Still, we refrain from attempting any attribution at a lower taxonomic level, as the material available lacks synapomorphies, pertaining to for example, the upper jaw, hyoid and gill arches, photophore pattern, and so on. (Fink & Weitzman, 1982; Harold & Weitzman, 1996; Harold, 1998). Stomiiforms were likely present since the Cenomanian, but the systematic placement of early representatives of this group (e.g., †*Paravinciguerria*) is still unclear (Arambourg, 1954; Carnevale & Rindone, 2011). If the eurytanian fossil is confirmed to be a crown stomiiform, and a gonostomatid by future fossil discoveries, this Paleocene occurrence will be the oldest for the family, followed by Eocene occurrences in Northern Italy (Giusberti et al., 2014) and Northern Caucasus (Prokofiev & Bannikov, 2002).

Teleostei indet. morphotype 5

Figure 8E

**Material.** AMPG_ATD_3a,b, very fragmentary dentary

**Description.** The dentary is higher posteriorly than anteriorly. Its posterior surface forms a deep indentation for the insertion of the anguloarticular. Anteriorly, a thin, recurved tooth issues near the symphysis. The overall appearance of this element is stockier than the stomiiform jaws described above.

**Remarks.** We note that such stocky jaws with recurved teeth are unknown in clupeids or stomiiforms. The hereby described jaw comes from a yet unknown taxon, but its incompleteness precludes further attribution.

## Discussion

Our paleontological excavations in the Maastrichtian and Paleocene fish-bearing horizons of Eurytania, constitute the first scientific collection efforts in over five decades (Renz, 1955; Koch & Nicolaus, 1969), and help shed light on the ichthyofaunal composition of the Tethys during the critical, but undersampled, time interval surrounding the deadly K–Pg extinction event. In this work, we report on a total of four localities, although additional, seemingly less productive localities have been recently discovered by one of us (T. Argyriou). Three of the newly reported localities (AND, SGL1, SGL2) are completely new, and yielded numerous partially articulated and disarticulated teleost remains from the late Maastrichtian. The fourth locality (ATD) is of Paleocene age and yielded fewer fossils, only one of which was articulated (see Clupeidae). ATD can be tentatively correlated with one of the previously reported localities (Koch & Nicolaus, 1969). A minimum of eight teleost taxa are represented in the material we collected from newly discovered late Maastrichtian localities (mostly from locality SGL1), assuming that the two †enchodontids are lumped into one taxon and that Teleostei indet. morphotype 3 can be treated collectively with Elopomorpha indet. However, the nature of the collected material is largely fragmentary and, in many cases, lacks definitive synapomorphies, leading us to treat it conservatively until future discoveries enable more detailed taxonomic analyses. The new faunal list for the Maastrichtian is as follows: Elopomorpha indet.; †Ichthyotringidae indet. sp. nov.; †Enchodontidae indet. 1; †Enchodontidae indet. 2; Eurypterygia indet.; and Teleostei indet. morphotypes 1–4.

**Figure 9:**
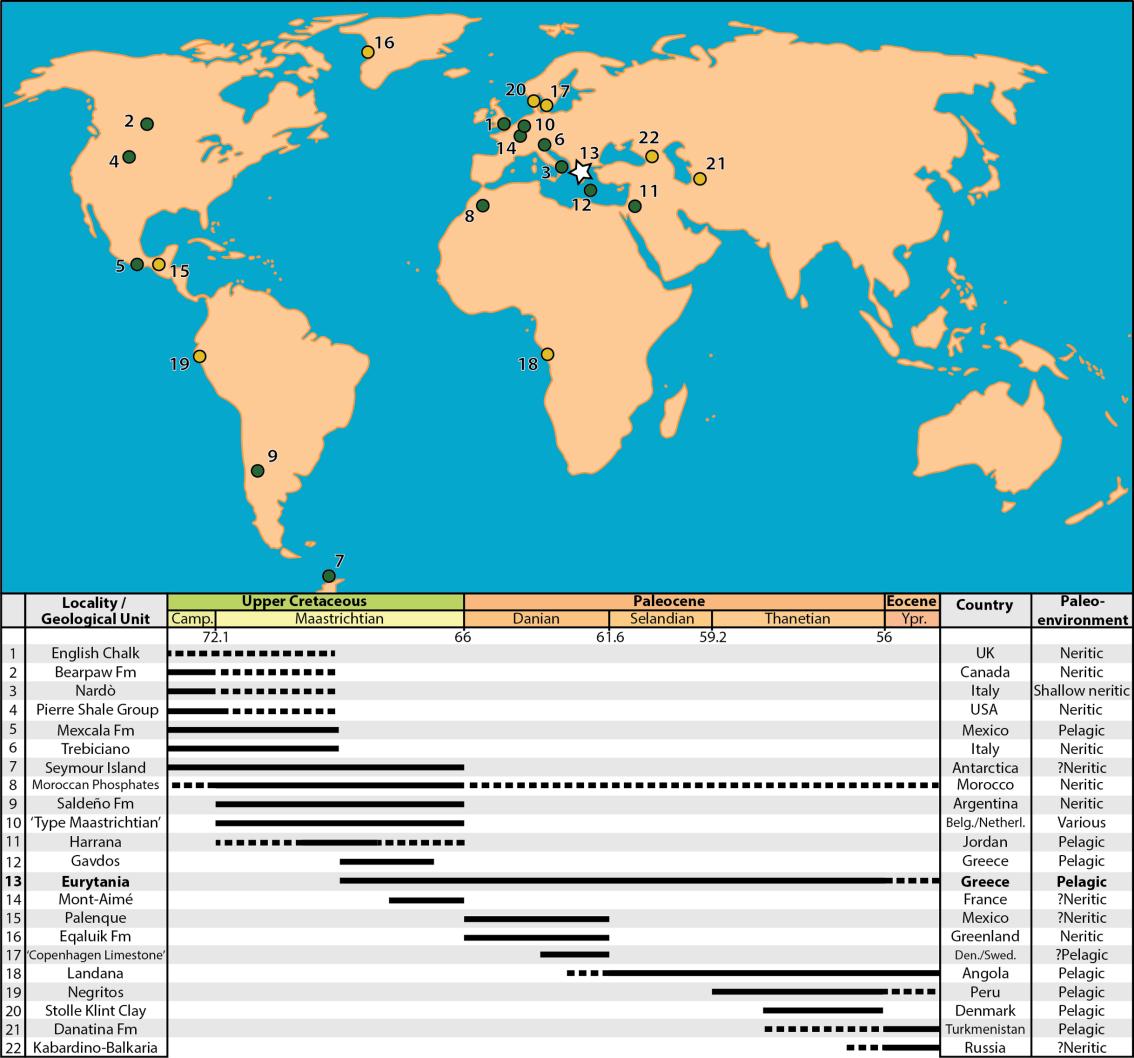
Map of the Maastrichtian (green dots) and Paleocene (yellow dots) faunas preserving marine actinopterygian fossils in anatomical connection. References for the faunas: (1) English Chalk, southern England, U.K. (Friedman et al., 2016); (2) Bearpaw Fm., St Mary River, Alberta, Canada (Newbrey & Konishi, 2015); (3) Nardò, Italy (Sorbini, 1981); (4) Pierre Shale Group, South Dakota, USA (Parris, Smith-Grandstaff & Gallagher, 2007); (5) Mexcala Fm., Guerrero, Mexico (Alvarado-Ortega et al., 2006); (6) Liburnica Fm., Trebiciano, Italy (Bannikov & Sorbini, 2000; Carnevale & Johnson, 2015; Taverne, Capasso & Arbulla, 2019); (7) López de Bertodano Fm., Seymour Island, Antarctica (Grande & Chatterjee, 1987; Cione et al., 2018); (8) Moroccan Phosphates (Arambourg, 1952; Bardet et al., 2017); (9) Saldeño Fm., Mendoza, Argentina (López-Arbarello, Arratia & Tunik, 2003); (10) “Type Maastrichtia”, Netherlands (Friedman, 2012); (11) Harrana, Jordan (Kaddumi, 2009; Lindgren, Kaddumi & Polcyn, 2013); (12) Pindos Unit, Gavdos Island, Greece (Cavin et al., 2012); (13) Pindos Unit, Eurytania, Greece (Koch & Nicolaus, 1969; this work); (14) Mont-Aimé, Champagne, France (Priem, 1898; Priem, 1908; Montenat et al., 2018); (15) Tenejapa-Lacandón Unit, Palenque, Chiapas, Mexico (Alvarado-Ortega et al., 2015); (16) Eqaluik Fm., Kangilia, Greenland (Capobianco, Foreman & Friedman, 2019); (17) Stevns Klint and København Limestone Fms., Stevns Klint and Faxe, Denmark/Limhamn, Sweden (Adolfssen, Milàn & Friedman, 2017); (18) Landana, Cabinda, Angola (Solé et al., 2019; Taverne et al., 2019); (19) Máncora Fm., Negritos, Peru (Friedman & Johnson, 2005); (20) Fur Fm., Stolle Klint Clay, Jutland, Denmark (Bonde, 1997); (21) Danatina Fm., Turkmenistan (Bannikov, 1993); (22) Abazinka Fm., Gerpegezh, Kabardino-Balkaria, Russia (Bannikov & Carnevale, 2012; Bannikov et al., 2017). Dotted lines represent weakly supported temporal range extensions.

A portion of our material cannot be attributed to known genera or species, which is however anticipated given the scarcity of data on late Maastrichtian teleosts in the global fossil record (Fig. 9). We note that our Teleostei indet. morphotype 4 likely comes from a previously unknown genus and species, as evidenced by more complete material from other Pindos Unit Maastrichtian exposures in Gavdos Island, Greece (T. Argyriou, 2019, personal observations). At the same time, the new †ichthyotringid fossils presented here possibly correspond to another undescribed species and extend the stratigraphic range of the lineage to the late Maastrichtian, adding it to the list of teleost casualties of the K–Pg Extinction. Similarly, Koch & Nicolaus (1969) hinted at an increased temporal range for †*Eurypholis*, but the presence of the genus in the Maastrichtian of Eurytania has not been verified by our collections. Similarly, we noted anatomical differences that did not allow us to readily place the new †enchodontid material in any known species, including the previously figured Eurytanian †*Enchodus* (Koch & Nicolaus, 1969), or †*Enchodus* cf. †*E. dirus* from the Maastrichtian of the Pindos Unit in Gavdos Island (Cavin, Alexopoulos & Piuz, 2012). Collectively, these observations are suggestive of additional undiscovered diversity within Late Cretaceous aulopiforms (see also Davis & Fielitz, 2010; Newbrey & Konishi, 2015). At the family level and at higher taxonomic levels, the Maastrichtian assemblages from Eurytania are dominated by faunal components, such as elopomorphs and epipelagic aulopiforms, typical of Late Cretaceous marine sites of the Tethys and beyond (Davis, 1887; Arambourg, 1952; Arambourg, 1954; Siegfried, 1954; Goody, 1969; Forey et al., 2003; Khalloufi, Ouarhache & Lelièvre, 2010; Friedman, 2012), while pelagic acanthomorph teleosts, which gradually rise to prominence in post-extinction faunas (Friedman, 2010; Near et al., 2013; Guinot & Cavin, 2016; Alfaro et al., 2018; Friedman et al., 2019), are seemingly absent (depending on the affinities of the unidentified eurypterygian). The overall character of the fauna points towards a higher-taxonomic and ecological continuum in global pelagic ecosystems spanning the entire Late Cretaceous.

Unlike previous reports from the area (Koch & Nicolaus, 1969), we only recovered one fragmentary chondrichthyan tooth (cf. Lamnidae), while †*Scombroclupea* (Clupeomorpha) or related clupeomorph material is absent from our Maastrichtian material, suggesting that these taxa were rare. At the same time, possible differences in the dentition between the putative †*Ichthyotringa* (=†*Rhinellus*) and †*Enchodus* material figured by Koch & Nicolaus (1969, Pl. 33:1,2*)*, and the material presented herein are noted. This could be explained by the likely presence of additional taxonomic diversity within the late Maastrichtian marly limestone horizons in the area. However, all recently collected †ichthyotringid dentigerous bones exhibit anteriorly inclined fangs, instead of the possibly vertically inclined ones of the specimen figured by Koch & Nicolaus (1969, Pl. 33:2*)*. This invites the second plausible explanation that some of the fossils reported by Koch and Nicolaus derive from slightly older, Late Cretaceous platy limestone facies, which are extensively exposed in roadcuts along the road from Aghia Trias to Domianoi, where the yielding locality (not yet rediscovered) is situated.

Actinopterygian fossils from the Paleocene of Eurytania seem to be scarce, and the slightly expanded faunal list is so far restricted to a minimum of three teleost taxa: Clupeidae indet.; Stomiiformes indet. and a possibly additional teleost taxon represented by a damaged jaw. Our Paleocene material does not contain any chondrichthyan elements, which have been previously reported from ATD (Koch & Nicolaus, 1969). Evidence available so far from the region (Koch & Nicolaus, 1969), combined with our observations on ATD, are indicative of rather oligospecific faunas and low overall abundance of fossil individuals. This is congruent with the common motif of Paleocene non-otolith marine fish-bearing sites, which as a rule comprise poorly preserved, low diversity fossil fish assemblages (e.g., Adolfssen, Milàn & Friedman, 2017; Schwarzhans & Milàn, 2017; Capobianco, Foreman & Friedman, 2019; Solé et al., 2019; Taverne et al., 2019; for an exception see Alvarado-Ortega et al., 2015). Still the potential of ATD should be noted, as it yields identifiable material like the Eurytanian clupeid, which is so far the oldest known from the Tethys, followed by the much better-preserved clupeids from the Ypresian of Bolca, Italy (Marramà & Carnevale, 2015a; Marramà & Carnevale, 2015b; Marramà & Carnevale, 2018). Similarly, the stomiiform from the site bridges a gap of over 40 Ma in the fossil record of this nowadays diverse group of deep-sea fishes (Prokofiev, 2005; Carnevale & Rindone, 2011).

### Paleoenvironmental and paleoecological signature of Maastrichtian fossil fish assemblages

The overall character of the sampled fossil assemblages in the vicinity of Karpenisi is congruent with an open, deep water depositional environment, also inferred by the platy limestone and calcareous marl sedimentation, which includes an abundance of planktonic foraminifera (Koch & Nicolaus, 1969; Fleury, 1980). Macroinvertebrates are generally scarce, with only poorly preserved †desmoceratid, or otherwise indeterminate ammonite shell traces found in SGL1 and nearby sites (Klug et al., 2020). Interestingly, several non-mineralized ammonite and coleoid jaws have been collected (Klug et al., 2020), exhibiting the rare potential of these transitional marly facies to preserve soft tissues, which can in turn be linked with low bottom oxygen levels and rapid burial. Low bottom oxygenation is further supported by the absence of benthic organisms in the sampled strata.

Much of the fish material corresponds to disarticulated or semi-articulated skeletal remains, which indicates some degree of transportation from their original habitats. The relative abundance and diversity of fossil aulopiforms can also be correlated with an open, deeper water setting. †Ichthyotringoids, for example, which are the most common teleosts in the Maastrichtian of Eurytania, have been reported from several older localities corresponding to open marine paleoenvironments (such as Jebel Tselfat; (Arambourg, 1954; Khalloufi, Ouarhache & Lelièvre, 2010), Hakel, Namura and Sahel Alma, Lebanon (Davis, 1887; Forey et al., 2003, and references therein), and Sendenhorst (Dietze, 2009)). Given their fossil record and their longirostrine anatomy, †ichthyotringids can be envisaged as Cretaceous epipelagic predatory analogs of modern needlefishes (Froese & Pauly, 2019). Many †enchodontids, like †*Enchodus*, are very common in Cretaceous open water deposits across the world and were seemingly able to cross oceanic basins (Arambourg, 1952; Goody, 1969; Cavin, Alexopoulos & Piuz, 2012; Da Silva & Gallo, 2016). The examined Maastrichtian fish-bearing horizons have not yielded fossils of deep bodied groups (e.g., †pycnodonts) and other bottom feeding or grazing fishes, which are more common in shallow water settings, suggesting that the “transitional facies to the flysch” of the Pindos Unit in Eurytania were not in the immediate paleogeographic vicinity of shallow reefal or epicontinental sea habitats (see Jebel Tselfat (Khalloufi, Ouarhache & Lelièvre, 2010), or Lebanese deposits (Forey et al., 2003) for examples, of more nearshore open water faunas containing possibly transported shallow water faunal elements). Macropredation is the main ecological theme suggested by the dentitions of most collected fossil fishes, and the presence of soft-bodied nektonic cephalopod remains in SGL1—as a possible source of food for fishes (Klug et al., 2020)—illuminate aspects of Maastrichtian food chain complexity in Eurytania. A synthesis of available fossil and lithological evidence points towards the presence of fully functional open marine ecosystems in the depositional environments of the Pindos Unit, and Tethys as a whole, right before the K–Pg Extinction event.

Scarcity of fossil evidence from Paleogene deposits in the area cannot yet support a detailed discussion on paleoenvironments, though clastic sedimentation, presence of plant debris (Koch & Nicolaus, 1969; Fleury, 1980; T. Argyriou and D. Davesne, personal observations at and near June 2019), in conjunction with mesopelagic organisms, such as stomiiforms (Froese & Pauly, 2019), are suggestive of closer proximity to the land with simultaneous preservation of unrestricted connection to open deep waters. The observed low diversity, low abundance character of the site might reflect the hypothesized, severely ecologically impoverished state of Tethyan marine ecosystems in the wake of the K–Pg Extinction (Sibert, Hull & Norris, 2014). However, additional exploration is required before completely ruling out any influence by local sedimentological and paleoenvironmental conditions.

### Nature of the Maastrichtian–Paleocene fossil record of actinopterygians

Marine vertebrate assemblages containing skeletal material in anatomical association from the time interval surrounding the K–Pg Boundary, although relatively rare in the global fossil record (Fig. 9), are crucial for attaining better-informed taxonomic, evolutionary and stratigraphically resolved perspectives on the extinction and, in conjunction with lithological and additional faunal traits of their host rocks, can help paint vivid pictures of ecosystemic changes through this major biotic crisis. Much of the current knowledge on the body-fossil record of Maastrichtian actinopterygians is based on adequately studied assemblages coming from the early part of the stage (e.g., the type Maastrichtian section in the Netherlands, Friedman, 2012), or sedimentary successions of largely Campanian age that range—sometimes putatively—into the early Maastrichtian (e.g., Nardò (Sorbini, 1981; Taverne, 2005; Friedman, 2009) and Trebiciano (Bannikov & Sorbini, 2000; Carnevale & Johnson, 2015)). The age of the locality of Mont-Aimé in the Paris Basin, which has yielded an articulated and exquisitely preserved, but low-diversity actinopterygian fauna of †pycnodonts and a percomorph—accompanied by abundant elasmobranch teeth and marine reptile remains (Priem, 1908; Montenat et al., 2018), has been recently pushed back from the Danian to the Maastrichtian (Guernet & Villier, 2017). It is worth noting that the actual fossiliferous horizon(s) that produced the fish fossils collected in the 1800s is nowadays unknown (Montenat et al., 2018). Numerous additional Maastrichtian localities of are scattered all over the world, but most of these correspond to non-bedded, high-energy deposits, which only yielded disarticulated fossils and are characterized by preferential preservation (e.g., winnowing)—and/or previous collection biases—towards large or robust elements and fossil individuals (Arambourg, 1952; Cappetta, 1972; Grande & Chatterjee, 1987; Prasad & Sahni, 1987; Chalifa & Lewy, 1991; Bardet et al., 2000; Murray, 2000; Alvarado-Ortega et al., 2006; Parris, Smith-Grandstaff & Gallagher, 2007; Friedman, 2009; Bardet et al., 2017; Cione et al., 2018). Identifiable actinopterygian components of such assemblages are usually dominated by isolated teeth, or dentigerous bones of typical late Mesozoic ray-finned fishes such as †ichthyodectoids or †enchodontoids. The global Paleocene fossil record is even poorer than that of the Maastrichtian, both in terms of number of known sites, but also in terms of recorded biodiversity and, in most cases, quality of preservation (Patterson, 1964; Thomas et al., 1999; Cavin, 2002; Friedman & Johnson, 2005; Parris, Smith-Grandstaff & Gallagher, 2007; Schwarzhans & Milàn, 2017; Capobianco, Foreman & Friedman, 2019). The Danian neritic sites near Palenque, Mexico, constitute notable exceptions from this rule (Alvarado-Ortega et al., 2015; Cantalice & Alvarado-Ortega, 2016; Cantalice, Alvarado-Ortega & Bellwood, 2020), yielding well-preserved and diverse shallow water actinopterygian assemblages comprising typical Paleogene (e.g., marine osteoglossomorphs) as well as derived faunal elements (including aulostomoids, pomacentrids and other percomorphs), which foreshadow the more modern-like, acanthomorph-dominated assemblages from the early Eocene of Turkmenistan and the Kabardino-Balkaria (Bannikov & Parin, 1997; Bannikov & Carnevale, 2012; Bannikov et al., 2017), Bolca, the London Clay and other localities (Arambourg, 1967; Daniltshenko, 1962; Eastman & Grande, 1991; Bannikov, 1993; Bonde, 1997; Afsari et al., 2014; Davesne, 2017; Beckett et al., 2018; Friedman & Carnevale, 2018).

The overall scarcity of information regarding this critical time interval appears exacerbated when examining the Maastrichtian fossil record of the Tethys. The Campanian–?Maastrichtian (Sorbini, 1981) of Nardò, Italy, yielded an enormous diversity of well-preserved, mostly neritic forms (e.g., Sorbini, 1981; Taverne, 2004; Taverne, 2005; the taxonomic diversity of Nardò is discussed and summarized in Friedman (2009)), comprising actinopterygians of different body sizes and phylogenetic affinities, and thus constituting an informative snapshot of Late Cretaceous Tethyan ecosystems. However, due to uncertainties regarding their geological age, Nardò fossils should better be excluded when discussing the status of Maastrichtian biodiversity (Friedman, 2009). Following the recent reappraisal of its age, the early Maastrichtian of Trebiciano, Italy, yielded articulated skeletons that add to our knowledge of the composition of shallow, or marginal, water faunas of Western Tethys (Bannikov & Sorbini, 2000; Carnevale & Johnson, 2015; Taverne, Capasso & Arbulla, 2019). The vast majority of remaining Tethyan assemblages exhibit a more offshore character and largely conform to the preservational and/or collection biases outlined above (e.g., Morocco, (Arambourg, 1952; Bardet et al., 2017); Negev Desert, Israel, (Chalifa, 1996; Chalifa & Lewy, 1991); Syria (Bardet et al., 2000); Jordan (Arambourg et al., 1959; Bardet & Pereda Suberbiola, 2002); Saudi Arabia (Kear et al., 2009)). Sorted, deeper water deposits such as those of the Moroccan or Middle Eastern phosphates sometimes exhibit three-dimensional fossil preservation, which largely applies to either just robust teeth (e.g., elasmobranchs or †enchodontoids), or isolated crania and vertebrae of large individuals. In such deposits, there is little probability for fossilization, retrieval, or positive identification of small sized and/or more delicately built fishes, like the putative eurypterygian presented here, or the small-sized †ichthyotringoid. Additionally, robust elements such as teeth can sometimes be reworked and re-deposited in younger horizons, confusing interpretations about potential K–Pg survivors (for some examples see Cavin, 2002). Two pelagic assemblages from the Maastrichtian constitute notable exceptions to the above; the ones presented here, and the assemblages of Harrana Formation in Jordan (Kaddumi, 2009). The Harrana fauna is associated with the deposition of phosphates in what is today the Middle East and North Africa, and has yielded abundant, seemingly articulated but largely overlooked teleost remains (†ichthyodectiforms, elopomorphs, aulopiforms, acanthomorphs) and other vertebrates from the (middle?, sensu Lindgren, Kaddumi & Polcyn, 2013) Maastrichtian (Kaddumi, 2009). Despite lagging behind in terms of taxonomic diversity, the preservation potential for delicate elements and organisms (see also Klug et al., 2020) of the Eurytanian assemblages resembles better that of the early Late Cretaceous Lagerstätten of the Western Interior Seaway (Shimada & Fielitz, 2006; Fielitz & Shimada, 2009) and Lebanon (Davis, 1887; Forey et al., 2003), rather than that of known assemblages from the Maastrichtian of the Tethys.

There is paucity of articulated marine actinopterygian specimens from the Paleocene of the “Mediterranean” portion of the Tethys. Few notable Tethyan fossiliferous localities and geological formations are associated with the Paleocene–Eocene thermal maximum (PETM), including: (i) the (?)Thanetian–Ypresian phosphate horizons in Tunisia, which yielded a single, incomplete menid (Astre, 1927; Friedman & Johnson, 2005); (ii) the sapropelitic layers of Kabardino-Balkaria, from which a centriscoid and a tetraodontoid were described (Bannikov & Carnevale, 2012; Bannikov et al., 2017), and (iii) the rich deposits of Turkmenistan, which host diverse, percomorph dominated assemblages of rather shallow water affinities, associated with the Eastern Tethys and the Paratethys (see for example, Bannikov & Parin, 1997). Although the first horizons might partially record the latter part of the Paleocene, the fish-bearing strata of the latter two were recently dated to the earliest Eocene (Bannikov & Carnevale, 2012; Bannikov et al., 2017). In this context, the well-stratified fossiliferous deposits of the Pindos Unit in Eurytania (Koch & Nicolaus 1969; this work) and Gavdos Island (Cavin, Alexopoulos & Piuz, 2012) are key for understanding the Tethyan pelagic biodiversity right before (late Maastrichtian) and after (Paleocene) the extinction event and—as demonstrated by the presence of †ichthyotringoids—can help ameliorate the associated Signor-Lipps Effect (Cavin, 2002) by preserving fossils from a wide range of body sizes, which co-existed in the same basin.

## Conclusions

After a hiatus of half a century, renewed fieldwork in the Cretaceous–Paleocene of the Pindos Unit in Eurytania, continental Greece, brought to light new localities and ray-finned fish assemblages from the Late Maastrichtian, and some additional fossils from the Paleocene. Despite the fragmentary state of preservation of the material, representatives of at least eight distinct morphotypes, belonging to up to seven different higher groups of teleosts (“families” or above) are recognized in the Maastrichtian, and a minimum of two morphotypes, belonging to two different groups, respectively in the Paleocene. Some of the recovered fossils (e.g., the †ichthyotringid, or the teleost indet. morphotype 4) exhibit characters that differentiate them from other Late Cretaceous taxa, hinting at the presence of more than one undescribed species in the Cretaceous of Eurytania. The Eurytanian assemblages constitute promising additions to the otherwise depauperate global fossil record of actinopterygians from the interval surrounding the K–Pg Extinction, and include small-sized taxa, such as the new †ichthyotringid, which are not represented—or not preserved—in better sampled penecontemporaneous localities (e.g., Moroccan phosphates, Arambourg, 1952). With an abundance of epipelagic Aulopiformes, the overall character of the Eurytanian Maastrichtian assemblages is similar to that of older Tethyan sites (see for example, the celebrated Lebanese deposits (Davis, 1887; Forey et al., 2003), or Jebel Tselfat(Arambourg, 1954)) and testifies to the presence of an ecological continuum in offshore marine actinopterygian faunas throughout the Late Cretaceous, thus, disproving notions of a staged decline of actinopterygian faunas immediately prior to the K–Pg Extinction. Collectively, the Late Maastrichtian vertebrate (this work) and invertebrate (Klug et al., 2020) fossils provide clues for fully functional offshore marine ecosystems, and conceivable food chains, of the Tethys, in close temporal proximity to the K–Pg extinction event. On the other hand, the Paleocene of Eurytania has so far produced very few fossils (Koch & Nicolaus, 1969; this work). Although this fact might reflect the depauperate state of post K–Pg Extinction faunas, a larger sample of fossils and layer-by-layer collecting is required to rule out potential collection or taphonomic biases. Still, our material includes a fragmentary stomiiform, which represents one of the earliest fossil representatives of the group.

Maastrichtian and Paleogene rocks are exposed over tens of km^2^ in Eurytania and further exploration and the ideal establishment of small-scale excavation quarries will doubtlessly lead to the discovery of many more fossiliferous sites and fossils with great prospects for better understanding the turnover in Tethyan actinopterygian faunas caused by the K–Pg Extinction and the recovery of post-extinction open water ecosystems. For example, better fossil sampling, also targeting otoliths and microvertebrate remains, of Eurytanian Paleogene horizons is amongst others anticipated to provide the means to address past hypotheses of severe Tethyan biotic depletion following the K–Pg Extinction (Sibert, Hull & Norris, 2014).We note that tracing the actual K–Pg boundary and attaining a better age resolution for Paleogene rocks in the area are still wanting before accurate correlations between Tethyan sites can be made. Future missions should also focus on the rediscovery of unmarked Maastrichtian horizons on the western slope of Nakena-Tsouma Ridge, which have previously yielded several fully articulated fish skeletons (Koch & Nicolaus, 1969), and which remained inaccessible during our missions, due to the lack of passable roads and the presence of dense forestation and fencing. Previous research also reported on the presence of a distinct calcareous fish-bearing horizon of possible early Eocene age within the flysch deposits (Koch & Nicolaus, 1969). This horizon remains completely unstudied from a vertebrate paleontological perspective. In addition to all the above, this work provides an early glimpse at the yet untapped potential informativeness of the Greek alpine sedimentary background, from the scope of vertebrate paleontology and evolution. Encouraged by new fossil findings from K–Pg of Eurytania (this work) and the Maastrichtian of Gavdos Island (Cavin, Alexopoulos & Piuz, 2012), we stress the need for further exploration in the Mesozoic and early Cenozoic sedimentary successions of the “External Hellenides” (see also Argyriou, in press).

## Additional Information and Declarations

### Field Study Permissions

The following information was supplied relating to field study approvals (i.e., approving body and any reference numbers): Permission for geological/paleontological fieldwork and museum collection work was given by the director of the Museum of Paleontology and Geology of the National and Kapodistrian University of Athens. All recovered fossils were properly deposited in the collections of the aforementioned institution.

### Data Availability

The following information was supplied regarding data availability: All specimens collected during our fieldwork in the K-Pg of Eurytania, including all specimens described in this work, are registered and stored in the collections of the Museum of Paleontology and Geology of the National and Kapodistrian University of Athens (AMPG). This is also true for all specimens previously on study loan to the MNHN, Paris, all of which have been returned to the AMPG. The list of described specimens is as follows for each taxonomic group: Elopomorpha: AMPG_SGL1_8, partial skull with attached mandible; AMPG_SGL1_25a,b lower jaw. Dercetidae: AMPG_SGL1_45, lower jaw Ichthyotringidae: AMPG_SGL1_1a,b partial skull and jaws; AMPG_SGL1_11, AMPG_SGL1_13 lower jaws; AMPG_SGL1_14a,b dermal palates and mandibles; AMPG_SGL1_23a,b anterior skull and hemimandible; AMPG_SGL1_26a,b, AMPG_SGL1_27 lower jaws; AMPG_SGL1_41a,b lower jaw; AMPG_SGL1_42a,b rostral fragments; AMPG_SGL1_53a,b,c; AMPG_SGL1_54a,b skull fragments. Enchodontidae 1: AMPG_SGL1_20, partial skull including mandibular and palatal elements; AMPG_SGL1_29a,b, fragmentary palate and suspensorium and lower jaws; AMPG_SGL1_30, anterior portion of skull including upper and lower jaws; AMPG_SGL1_33, slab bearing anguloarticular and palatine bones; AMPG_SGL2_1, jaw fragment Enchodontidae 2: AMPG_SGL1_2a–c, partially disarticulated skeleton Eurypterygia indet: AMPG_AND_2a,b, partially disarticulated individual Teleostei morph 1: AMPG_SGL1_5, tooth crown Teleostei morph 2: AMPG_SGL1_10a,b, slab with isolated dentary; AMPG_SGL1_32, slab with two dentaries Teleostei morph 3: AMPG_AND_6, left maxilla Teleostei morph 4: AMPG_SGL1_15a,b, dentigerous bone; AMPG_SGL1_48, dentigerous bone Clupeidae: AMPG_ATD_2a,b, part and counterpart of caudal region and fin Stomiiformes: AMPG_ATD_1a,b, part and counterpart of left and right lower jaws.
